# Identification and exploration of pharmacological pyroptosis-related biomarkers of ulcerative colitis

**DOI:** 10.3389/fimmu.2022.998470

**Published:** 2022-10-13

**Authors:** Kaiwei Chen, Shipeng Shang, Shengnan Yu, Luwen Cui, Shangyong Li, Ningning He

**Affiliations:** School of Basic Medicine, Qingdao Medical College, Qingdao University, Qingdao, China

**Keywords:** ulcerative colitis, pyroptosis, transcriptome, IL1b, DSS-induced colitis

## Abstract

Ulcerative colitis (UC) is a chronic inflammatory bowel disease (IBD). Its etiology is unclear. Much evidence suggests that the death of abnormal intestinal epithelial cells (IECs) leads to intestinal barrier disruption, and the subsequent inflammatory response plays a vital role in UC. Pyroptosis is a form of programmed inflammatory cell death, and the role of pyroptosis in UC etiology remains to be explored. This study identified 10 hub genes in pyroptosis by gene expression profiles obtained from the GSE87466 dataset. Meanwhile, the biomarkers were screened based on gene significance (GS) and module membership (MM) through the Weighted Gene Co-Expression Network Analysis (WGCNA). The following analysis indicated that hub genes were closely associated with the UC progression and therapeutic drug response. The single-cell RNA (scRNA) sequencing data from UC patients within the GSE162335 dataset indicated that macrophages were most related to pyroptosis. Finally, the expression of hub genes and response to the therapeutic drug [5-aminosalicylic acid (5-ASA)] were verified in dextran sulfate sodium (DSS)-induced colitis mice. Our study identified *IL1B* as the critical pyroptosis-related biomarker in UC. The crosstalk between macrophage pyroptosis and IEC pyroptosis may play an essential role in UC, deserving further exploration.

## Introduction

Ulcerative colitis (UC) is a chronic inflammatory bowel disease (IBD) of unclear etiology, beginning in the rectum and subsequently spreading to the colonic mucosa (1). The incidence rate of UC is 9 to 20 cases per 100,000 person-years, and the prevalence rate is 156 to 291 cases per 100,000 people, constituting a global burden (2). The quality of life of UC patients is commonly impaired due to diarrhea, abdominal pain, bloody stools, and the elevated risks of colon and rectal cancer (3). Various factors have been implicated in developing UC, such as dysregulated immune response, gut microbial dysbiosis, genetic susceptibility, and environmental influences (4). UC patients cannot be wholly cured and often take long-term medication (5). There is a widely accepted theory that a vicious cycle of intestinal barrier disruption, cell death, and subsequent inflammatory response rests at the heart of chronic inflammation (6).

The continuous monolayer of IECs, the first barrier against microbial and environmental pressure, implements a critical innate immune function (7). Remarkably, the abnormal death of large numbers of IECs is observed in preclinical models of UC patients (8). Moreover, intestinal barrier dysfunction is detected by confocal endoscopy before the occurrence of intestinal lesions, thereby predicting the recurrence of IBD (9). Pyroptosis, also called inflammatory cell necrosis, is a new form of programmed inflammatory cell death (10). The canonical pyroptosis pathway depends on caspase-1 for the cleavage of gasdermin D (GSDMD). In contrast, the non-canonical pathway relies on caspase-4/5/11. After cleavage, the N-terminal end of GSDMD develops a transmembrane pore, releasing inflammatory cytokines such as IL-1β and IL-18, and interferes with ion and water regulation, ultimately causing intense inflammation and cell death (11). This pattern of cell death accompanied by a robust inflammatory response becomes a double-edged sword for the host (10). An over-activated inflammatory response helps the host defend against pathogenic infections but could also cause various inflammatory diseases, including sepsis (12) and gout (13).

During the active phase of UC, several different forms of cell death, including pyroptosis, are activated (14). The development of UC could be related to pyroptosis of IECs and the release of inflammatory factors. The relationship between the two has attracted significant interest (15). For instance, IL-36β, a member of the IL-36 subfamily of the IL-1 family, increases the pathology of DSS-induced colitis in mice by enhancing Th2 responses in LPL while decreasing Foxp3+ Treg responses (16). However, current molecular mechanisms associated with pyroptosis in UC are lacking in research. Therefore, exploring its regulatory mechanisms and gene expression characteristics will help understand the etiology of UC and provide a new perspective on UC treatment.

In this study, hub genes associated with pyroptosis in UC were identified by Weighted Gene Co-Expression Network Analysis (WGCNA) combined gene expression matrix using the GSE87466 dataset as the base data. After validation by GSE92415, GSE107499, GSE59071, GSE73661, and GSE46451 datasets, the expression change of hub genes was observed after different drug treatments to demonstrate the role of pyroptosis in UC. Subsequently, we explored the expression patterns of hub genes in the macrophage clusters associated with pyroptosis of immune cells by single-cell RNA analysis of the GSE162335 dataset. We identified macrophage clusters having different roles in UC pyroptosis to reveal the mechanisms related to pyroptosis in UC.

## Materials and methods

### Datasets and preprocessing

Gene expression profiles and corresponding clinical data of UC were retrieved from the Gene Expression Omnibus (GEO) database (http://www.ncbi.nlm.nih.gov/geo). Information on all the datasets in this study is described in **Table 1**. The patient demographic characteristics for all the datasets are available in Supplementary Material 1. Pyroptosis-related genes (PRGs) were procured from the UniProt database (https://www.uniprot.org/), MSigDB database (https://www.gsea-msigdb.org/), and previous studies in the literature (17, 18) (**Supplementary Table S1**).

**Table 1:** Information for all the datasets in this study.

Dataset	Platform	Title
GSE87466	GPL13158	[HT_HG-U133_Plus_PM] Affymetrix HT HG-U133+ PM Array Plate
GSE92415	GPL13158	[HT_HG-U133_Plus_PM] Affymetrix HT HG-U133+ PM Array Plate
GSE107499	GPL15207	[PrimeView] Affymetrix Human Gene Expression Array
GSE59071	GPL6244	[HuGene-1_0-st] Affymetrix Human Gene 1.0 ST Array
GSE73661	GPL6244	[HuGene-1_0-st] Affymetrix Human Gene 1.0 ST Array
GSE46451	GPL10558	Illumina HumanHT-12 V4.0 expression beadchip
GSE162335	GPL20301	Illumina HiSeq 4000

### Differential expression analysis

The dataset GSE87466 containing 87 UC patients and 21 healthy individuals was utilized to identify the differentially expressed genes (DEGs) between the UC and healthy controls (19). The DEGs were screened having a threshold of *p*-value < 0.05 and |log2FC| > 1 by using the “limma” package. DEGs were visualized using the R packages “ggplot2” and “pheatmap.”

### Construction of co-expression network and identification of modules

The 3,945 variant genes were screened (**Supplementary Table S2**) with a coefficient of variation > 0.08 to reduce the computational effort of the whole network and maintain the characteristics of a scale-free topological network structure. A weighted gene co-expression network was constructed with the R package “WGCNA” for the 3,945 variant genes. First, the sample data with abnormal gene expression values were filtered using hierarchical clustering, and the “pickSoftThreshold” function was utilized to assess the appropriate soft threshold *β*. Subsequently, the Pearson correlation coefficients of the gene were determined, the weighted adjacency matrix was constructed using a soft threshold β of 13, and the adjacency matrix was converted into the topological overlap matrix (TOM). The minimum module size cutoff was set to 30, and the same modules were merged using a threshold value of 0.2. Genes with similar expression patterns were grouped within the same module. Module eigengene (ME) was calculated to represent the overall gene expression level inside the module and used to identify modules significantly correlated with the disease.

### Identification of pyroptosis-related hub genes and biomarkers

The hub gene was retrieved by taking the overlapping PRGs, DEGs, and genes from the module having the highest relevance to UC in WGCNA. Biomarkers were screened from hub genes depending on the criteria of gene significance (GS) > 0.2 and module membership (MM) > 0.8 in the WGCNA (20). The receiver operating characteristic (ROC) analysis of the hub genes was performed with the R package “pROC.”

### Immune cell infiltration estimation

The relative abundance of immune cells within the colonic mucosal tissue of the UC and healthy controls was assessed using the CIBERSORT algorithm (https://cibersort.stanford.edu/) (21). The difference between the two groups of immune cells was compared with Student’s *t*-test and visualized by a “ggboxplot.” The correlation between each infiltrating immune cell and the relationship between hub genes and immune cells was visualized using the “corrplot” package.

### Single-cell analysis

The dataset GSE162335 contained single-cell sequencing data of immune cells from the CD45+ colonic lamina propria of the 11 UC patients (22). The “Seurat” package was utilized for subsequent data processing (23). The low-expressing cells and genes were filtered while ensuring that the percentage of mitochondria per cell was below 5% and the features of genes were below 6,000 and above 500. Finally, a total of 18,375 cells were identified. After normalization using the “LogNormalize” method, 3,000 highly variable genes (HVGs) were identified with the “vst” method. PCA was applied to identify significant principal components based on the expression of HVGs. Thus, 25 PCs were selected for t-SNE analysis having a resolution of “2” to identify the different clusters. “FindAllMarkers” function (logfc.threshold = 0.25) was incorporated to identify DEGs in each cluster and marker gene for each cluster using avg_log2FC>1.

Using the “BlueprintEncodeData” dataset from the R package “celldex” as a reference, each cluster was initially annotated with “SingleR.” The R package “scHCL” was used to annotate the cluster of interest (24) using the Human Cell Landscape (http://bis.zju.edu.cn/HCL/) and the scRNASeqDB (https://bioinfo.uth.edu/scrnaseqdb/) databases as the secondary reference.

The R package “AUCell” was used to evaluate the response of single cells to PRGs (25), and the “Aucell_explorethreshold” function was used to determine the threshold for identifying gene set active cells. Then, the AUC score of each cell was mapped to the t-SNE embedding with “ggplot2” for visualization. The “Monocle” package was utilized for cell pseudotime analysis (26).

### Functional annotation and pathway enrichment analysis

GO annotations of genes from the R package “org.Hs.eg.db” (version 3.1.0) were utilized as background. The selected genes were mapped to the background set, and the enrichment analysis was performed using the R package “clusterProfiler” (version 3.14.3). *p* < 0.05 and FDR < 0.25 were considered statistically significant.

### Animal experiment

The experimental mice and standard rodent chow food were obtained from Jinan Pengyue Laboratory Animal Breeding Company (Jinan, China). The experimental procedures were approved by the Ethics Committee of the Medical College of Qingdao University (QDU-AEC-2022314). C57BL/6J mice (18–20 g) were randomized into three groups (*n* = 6) after 1 week of acclimation. NC group: no extra treatment for 7 days; DSS group: free drinking water containing 2.5% DSS during 7 days; 5-aminosalicylic acid (5-ASA) group: the free drinking water with 2.5% DSS for 7 days while 5-ASA (400 mg/kg/day) was administered by gavage. At the end of the experiment, the entire colon was excised, and the colon length was determined. The collected colon tissue was stored at −80°C for analysis.

### Histopathological analysis

The collected colonic tissues were fixed in 10% formalin overnight. The fixed tissues were embedded in paraffin and sliced in gradient concentrations of alcohol after dehydration at a thickness of 5 μm. They were then stained with hematoxylin and eosin (H&E) and observed with a 200× magnification (E100, Nikon, Tokyo, Japan) and an imaging system (DS-U3, Nikon, Tokyo, Japan). As previously described, the histological score for H&E staining was assessed in a blinded fashion (27).

### RNA extraction and quantitative real-time PCR analysis

The extraction of total RNA and reverse transcription were performed based on the kit instructions of SparkJade (Jinan, China). The RT-qPCR primers are depicted in **Supplementary Table S3**. The gene expression levels were normalized with GAPDH, and the relative quantification of gene expression was determined using the 2^−ΔΔCt^ method.

### Enzyme-linked immunosorbent assay

At the end of the experiment, mice were anesthetized with chloral hydrate and separated from the serum by centrifugation (3,500 rpm, 4°C, 30 min) after picking out the eyeball. The ELISA kit was purchased from ABclonal (#RK00027, Wuhan, China). The serum levels were measured based on the instruction of the ELISA kit.

### Statistical analysis

All the statistical analyses were performed using the R software (version 4.1.1). All the data are expressed as mean ± SE. Unpaired Student’s *t*-test was used to compare two groups, and one-way ANOVA was used for three and more group comparisons. Tukey HSD was used for inter-comparison between multiple groups. This statistical analysis was performed using the R package “ggpubr” and “stats.” *p* < 0.05 was considered statistically significant.

## Results

### Research design summary

**Figure 1:**
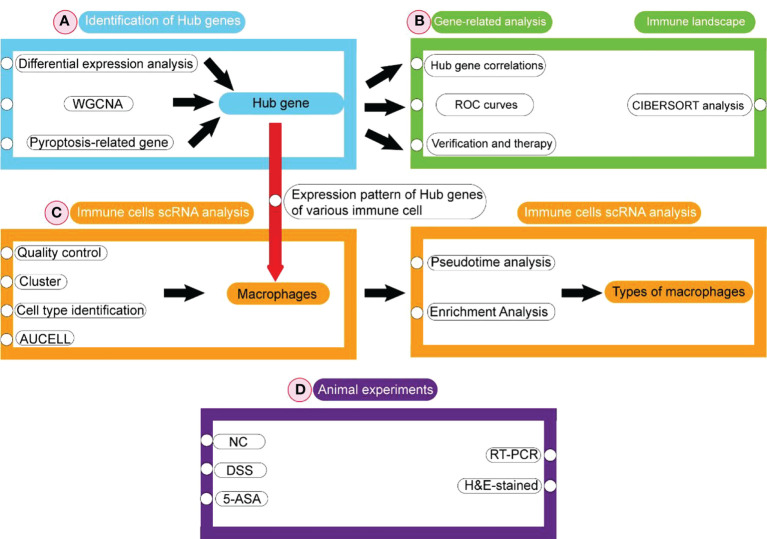
Flowchart of bioinformatics analyses in this study.

The flowchart is shown in **Figure 1**. Firstly, DEGs were screened between the UC patient and the healthy controls from the GEO database. Subsequently, pyroptosis-related hub genes were identified through DEGs combined with genes of the key module in WGCNA and PRGs. Based on GS and MM values, six genes were screened as biomarkers from the hub genes. ROC analysis was used to determine their diagnostic values. The obtained hub genes were validated from two aspects. Next, the differences in hub genes between UC/healthy controls, mucosal lesional/nonlesional group, and active/inactive/healthy controls were validated depending on three datasets obtained from the GEO database. The pattern of changes in the hub gene after treatment with 5-ASA, IFX (infliximab), and VDZ (vedolizumab) was assessed. Infiltrating immune cells in UC patients were analyzed with CIBERSORT. Finally, the expression pattern of hub genes was evaluated in immune cells from the colonic lamina propria of UC patients at the single-cell level. The hub gene expression was verified using animal experiments.

### Identification of DEGs and functional annotation and pathway enrichment of DEGs

**Figure 2:**
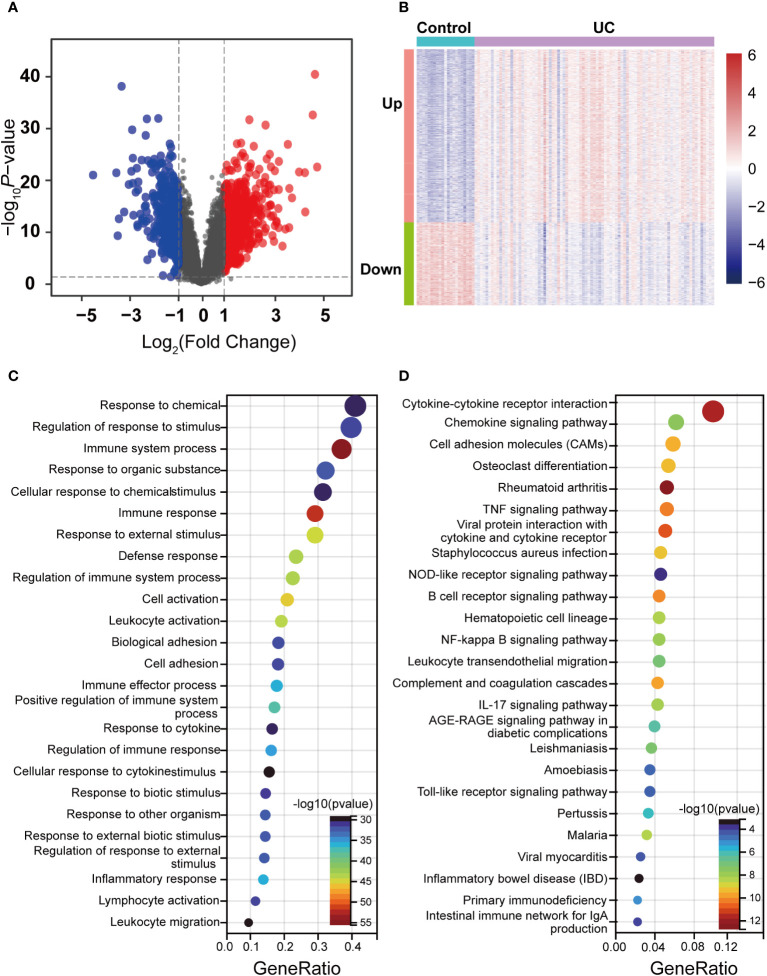
Identification of DEGs between the UC and control groups in the dataset and enrichment analysis. **(A)** Volcano maps of DEGs between UC and control, where red dots represent the upregulated genes, blue dots represent the downregulated genes, and gray dots represent no differential gene. **(B)** Heatmap of DEGs in UC and control. Red indicates high expression, and green indicates low expression. **(C)** GO enrichment analysis of DEGs. **(D)** KEGG pathway enrichment analysis of DEGs.

Correlation analysis of the GSE87466 dataset revealed stronger intra-group correlations for the UC group (**Figures S1A, B**). We identified 1,247 DEGs, including 843 upregulated and 404 downregulated genes, using *p*-value < 0.05 and |log2FC| > 1 as the threshold (**Supplementary Table S4**). The up- and downregulation distributions of DEGs were shown in the volcano plot (**Figure 2A**). Heatmap showed the expression patterns of DEGs and relative consistency within groups. Upregulated DEGs showed a positive correlation with the UC group and a negative correlation with healthy controls, while downregulated DEGs revealed the opposite (**Figure 2B**).

GO and KEGG enrichment analysis was performed to understand the functions of DEGs better. GO terms of biological process (BP) described that DEGs were primarily enriched in immunomodulation and immune response, such as response to chemical and immune system processes (**Figure 2C**). Moreover, the enriched KEGG pathway showed that a series of pathways associated with the inflammatory response was activated, such as Cytokine–cytokine receptor interaction, the B-cell receptor signaling pathway, the Chemokine signaling pathway, and the IL-17 signaling pathway (**Figure 2D**). Notably, DEGs were also enriched by pyroptosis-related inflammatory pathways, including the NOD-like receptor signaling pathway, the NF-kappa B signaling pathway, the Toll-like receptor signaling pathway, and the TNF signaling pathway. The enrichment results of these DEGs revealed that various external stimuli induced a series of immune responses, such as activating some inflammatory pathways associated with pyroptosis, indicating that pyroptosis is widely activated in UC. Moreover, the enrichment results of these DEGs also predicted a damaged intestinal barrier among UC patients. Intestinal barrier function has three main components: mechanical, ecological, and immune. The disruption of the immune barrier often leads to functional impairment of the other two. As mentioned above, these terms focusing on immune responses and inflammatory pathways indicate that the immune barrier is severely impaired among UC patients. This damage further disrupts the mechanical barrier, including the abnormal death of IECs.

### WGCNA construction and key modules identification

**Figure 3:**
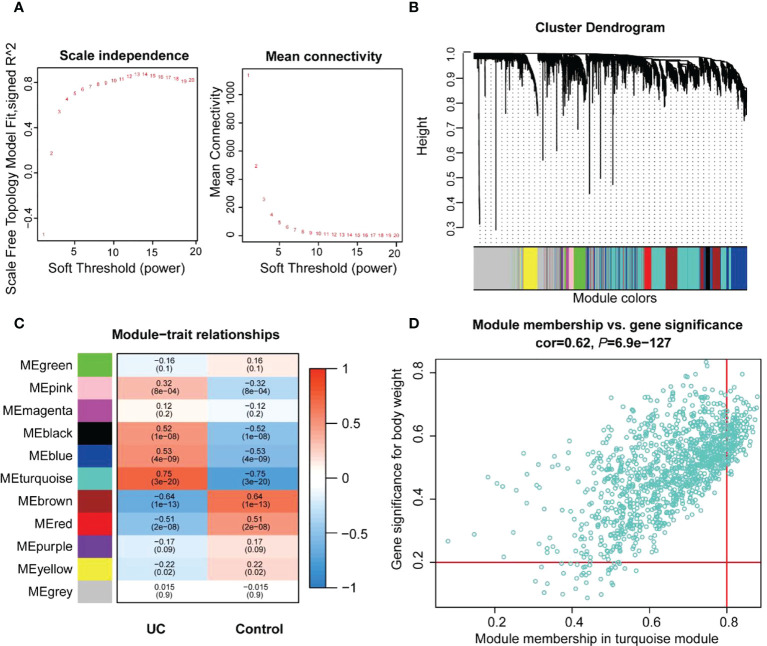
Identification of the key modules and genes associated with UC by WGCNA. **(A)** Estimating the scale independence index of the 1–20 soft threshold power and determining the mean connectivity of the 1–20 soft threshold power. **(B)** Module clustering dendrogram derived from the 1-tom matrix. The different color bands represent different modules. **(C)** Correlations between the various modules and traits. The number of each module depicts the correlation coefficient with the trait, and the color of the module ranges from red to green, showing high to low correlation. **(D)** Scatter plot of the turquoise module genes. The vertical coordinate represents the GS score, and the horizontal coordinate represents the MM score for each gene.

The co-expression network was developed to identify the most relevant modules for UC based on the expression of 3,945 CV genes. The 108 samples were clustered, and the two outlier data were removed (**Figure S1C**). A scale-independent topological network (soft threshold 13 scale-free *R* ^2^ 0.87) and the mean connectivity network were established (**Figure 3A**). We obtained 11 gene modules through hierarchical clustering and module merging (**Figure 3B**). Among these, black (*r* = 0.52, *p* = 1e-8), blue (*r* = 0.53, *p* = 4e-9), and turquoise (*r* = 0.75, *p* = 3e-20) modules were significantly correlated with UC (**Figure 3C**). The turquoise module, containing 1,186 genes, was selected as the feature module for UC depending on the correlation coefficient and *p*-value (**Supplementary Table S5**). Thus, 354 genes were screened as module hub genes from the turquoise module depending on GS > 0.2 and MM > 0.8 (**Figure 3D**).

### Identification of pyroptosis-related hub genes and biomarkers

**Figure 4:**
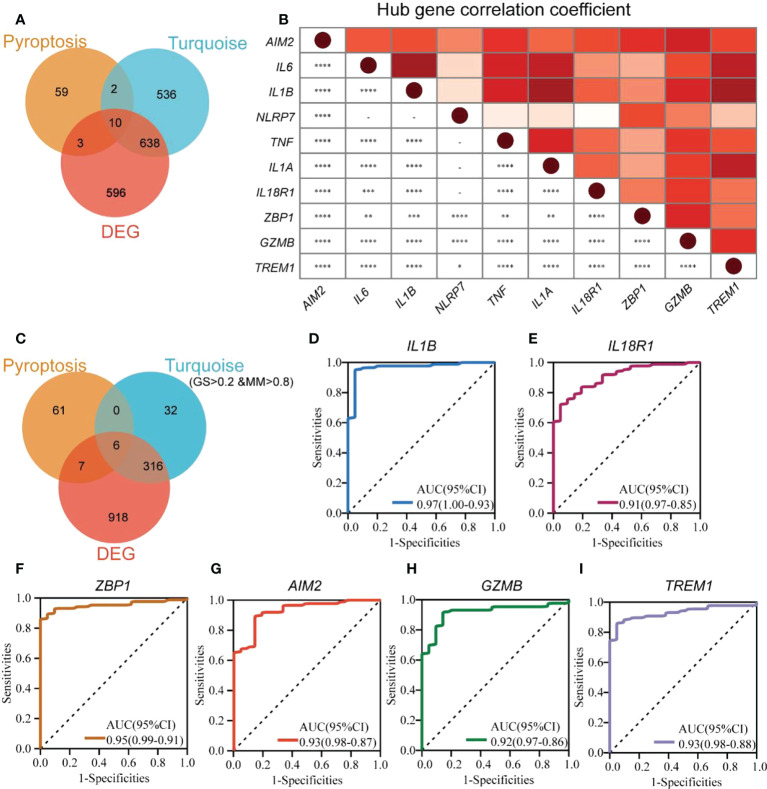
Identification of pyroptosis-related hub genes and biomarkers in UC. **(A)** The Venn diagram shows the overlapping genes generated by the intersection of turquoise module genes, DEGs, and PRGs. **(B)** Expression correlation matrix of each PRG hub gene in UC. **(C)** The Venn diagram shows the overlapping genes generated by the intersection of turquoise module hub genes, DEGs, and PRGs. ROC curves for *IL1B* **(D)**, *IL18R1* **(E)**, *ZBP1* **(F)**, *AIM2* **(G)**, *GZMB* **(H)**, and *TREM1* **(I)**. *p < 0.05, **p < 0.01, ***p < 0.001, and ****p < 0.0001.

We performed GO enrichment analysis to analyze 74 PRGs and observed that these genes were enriched with interleukin-1-related biological processes along with pyroptosis (**Figure S2**). This indicated that pyroptosis could be closely associated with interleukin 1. The 10 hub genes (*AIM2*, *IL6*, *IL1B*, *NLRP7*, *TNF*, *IL1A*, *IL18R1*, *ZBP1*, *GZMB*, and *TREM1*) were obtained from the overlap of DEGs, PRGs, and turquoise module genes (**Figure 4A**). *NLRP7* and *ZBP1* were poorly interrelated with other hub genes, and *IL1B* had the highest correlation with *IL6*, *IL1A*, and *TREM1*, depending on the expression patterns of the hub genes (**Figure 4B**).

To identify new UC biomarkers related to pyroptosis, the overlapping parts of turquoise module hub genes, DEGs, and PRGs were taken as new biomarkers (**Figure 4C**). A total of six genes (*IL1B*, *IL18R1*, *ZBP1*, *AIM2*, *GZMB*, and *TREM1*) were obtained. ROC analysis was performed to verify the diagnostic significance of the six biomarkers (**Figures 4D–I**). The AUC values for all the genes were more significant than 0.75, with *IL1B* (AUC 0.97, 95% CI) with the largest AUC value and *IL18R1* (AUC 0.91, 95% CI) showing the smallest AUC value. This indicated that *IL1B*, *IL18R1*, *ZBP1*, *AIM2*, *GZMB*, and *TREM1* could be new UC biomarkers associated with pyroptosis.

### Verification of pyroptosis-related hub genes

**Figure 5:**
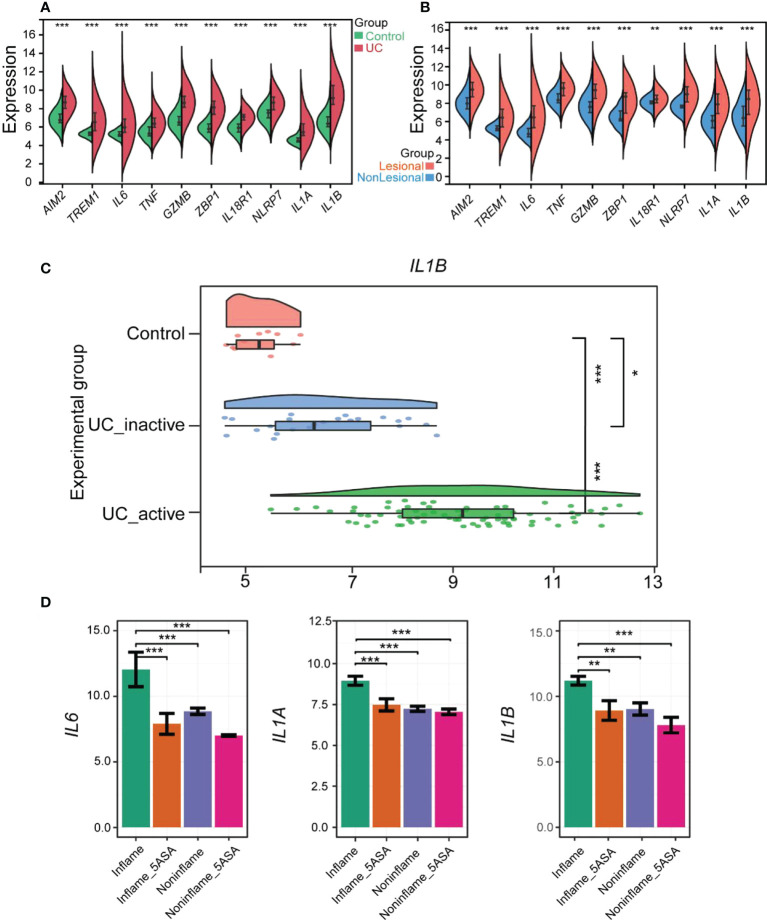
Verification of PRGs hub genes in UC and Evaluation of 5-ASA for treating UC pyroptosis. **(A)** The violin plot shows the expression of 10 hub genes in the colonic mucosa of the UC group and the control group in GSE92415. **(B)** Comp violin plot shows the expression of 10 hub genes in the lesional and nonlesional colonic mucosa of UC patients in GSE107499. **(C)** The raincloud plot shows the expression of IL1B in the colonic mucosa of control, inactive, and active UC patients in GSE59071. **(D)** 5-ASA alleviates rectal mucosal damage in UC patients by controlling *IL6*, *IL1A*, and *IL1B* in the RPGs hub genes. The relative expression levels of *IL6*, *IL1A*, and *IL1B* within the rectal mucosa of inflame (inflammation but no drug), inflame_5-ASA (inflammation and treated with 5-ASA), non-inflame (non-inflammation and no drug), and non-inflame_5-ASA (non- inflammation and treated with 5-ASA). *p < 0.05, **p < 0.01, and ***p < 0.001.

The 10 hub genes were validated using the GSE92415 dataset (27). All the hub genes were significantly different in UC and healthy controls (*p* < 0.001, **Figure 5A**). Moreover, all the hub genes were significantly different between lesional and nonlesional groups from the GSE107499 dataset (**Figure 5B**).

In addition, we compared the differences in hub genes between activated UC groups, inactivated UC groups, and healthy controls by the GSE59071 dataset (28). Only IL1B showed a statistical difference among the activated UC groups, inactivated UC groups, and healthy controls (**Figure 5C**). All the hub genes except *IL1B* were not significantly different in healthy controls and inactivated UC groups included *IL6* and *TNF*, common pro-inflammatory factors. Subsequently, four other pro-inflammatory factors, including *IL8*, *IL17A*, *IL18*, and *IL33*, were explored, which were thought to be closely associated with the development of UC disease. However, their role in pyroptosis remains unexplored. The expression pattern of *IL33* was similar to *IL1B*, with significant differences between all three groups (**Figure S3**). The relationship between *IL33* and UC could also be focused on in the future (29). Therefore, *IL1B* was the more vital of the hub genes and strongly correlated with other hub genes.

### Drugs improve rectal and colonic mucosal damage in UC patients by reducing pyroptosis-related hub genes

**Figure 6:**
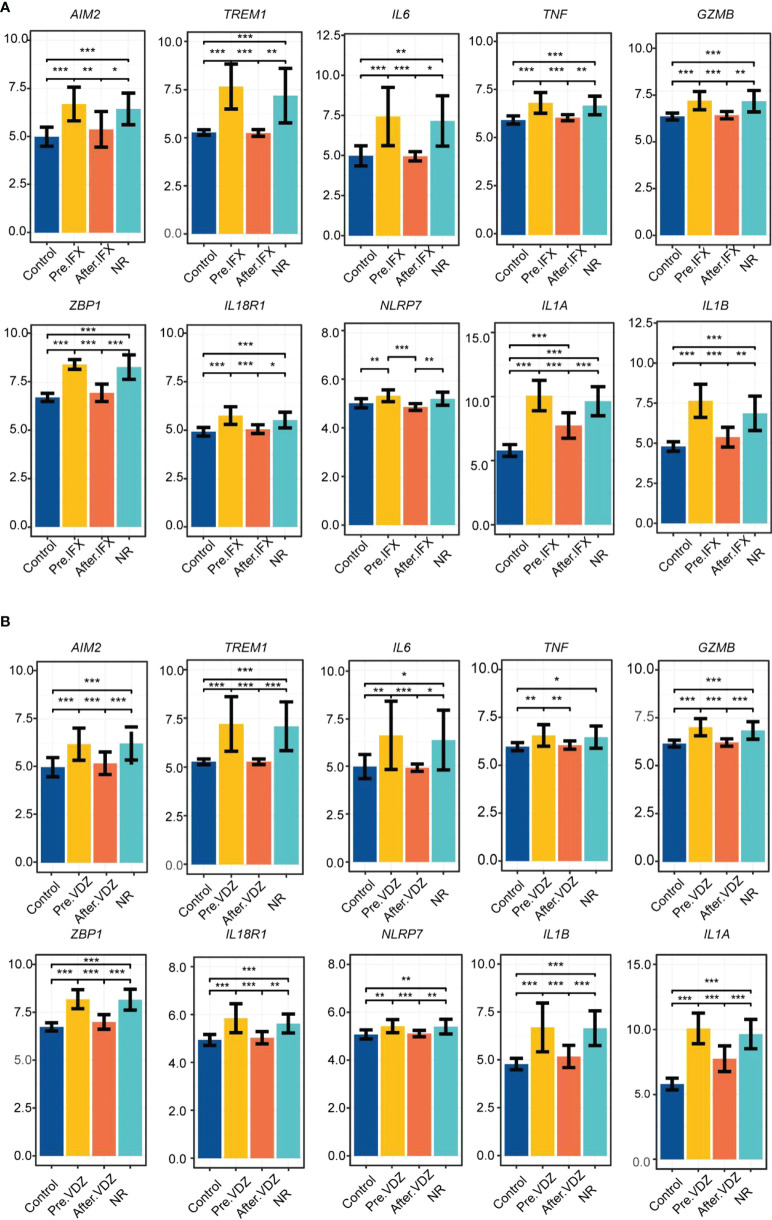
IFX and VDZ reduce impaired colonic mucosa of UC patients by regulating the PRGs hub gene. **(A, B)** The relative expression levels of *AIM2*, *TREM1*, *IL6*, *TNF*, *GZMB*, *ZBP1*, *IL18R1*, *NLRP7*, *IL1A*, and *IL1B* in the colonic mucosa of control, Pre.IFX (UC patients before IFX therapy), After.IFX (UC patients in remission after IFX therapy), and NR (UC patients not responding to IFX therapy). *p < 0.05, **p < 0.01, and ***p < 0.001.

Subsequently, the influence of drugs on changes in the expression of pyroptosis-related hub genes was explored using GSE46451 and GSE73661 (30). 5-ASA was the drug of choice for mild-to-moderate UC (31). IFX, adalimumab, and golimumab that target TNF-α, VDZ that target α4β7 integrin, and ustekinumab that target IL-12 and IL-23 are the five most common biologics approved for treating UC, and they were recommended as the first-line treatment for moderate-to-severe UC (32). It revealed no difference in the expression of *AIM2*, *TREM1*, *TNF*, *GZMB*, *ZBP1*, *IL18R1*, and *NLRP7* before and after 5-ASA effect on inflamed or non-inflamed rectal mucosa of UC patients *in vitro*, indicating that 5-ASA may not affect these seven RPGs (**Figure S4**). However, the expression levels of *IL6*, *IL1A*, and *IL1B* were significantly decreased in the inflamed rectal mucosa after receiving 5-ASA. These three genes also depicted significant differences between inflamed and non-inflamed rectal mucosa (**Figure 5D**). Meanwhile, the expression of hub genes in the colonic mucosa of patients with active UC significantly decreased after IFX treatment. Moreover, no difference between pre-IFX treatment and non-responders but a significant difference was observed between after-IFX treatment and non-responders. Most importantly, the expression levels of the other nine hub genes were restored to the levels of healthy controls, except for *IL1A* (**Figure 6A**). The expression patterns of pyroptosis-related hub genes before and after VDZ treatment of patients with active UC were similar to IFX (**Figure 6B**). Therefore, we speculated that IFX and VDZ could have reduced the occurrence of pyroptosis while treating UC. 5-ASA has been used to treat mild UC for nearly three decades, while biologics such as IFX and VDZ were often used to treat patients with moderate and severe UC. Based on the abovementioned pattern of hub genes, we speculated that the progression of the UC condition could be associated with pyroptosis.

### Immune infiltration analysis

**Figure 7:**
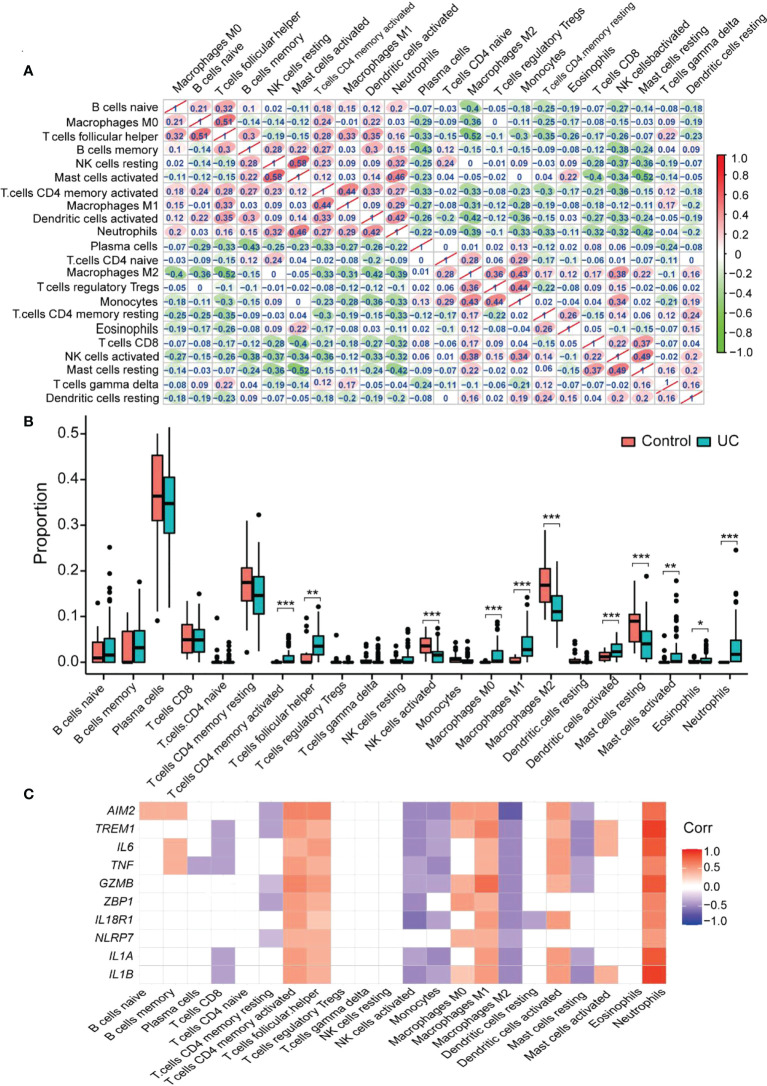
CIBERSORT probes the immune-infiltration landscape of UC. **(A)** The interrelationship between different infiltrating immune cells. Red to green indicates the change from high to low correlation. **(B)** Boxplot shows the colonic immune cell infiltration difference between the control and UC groups. Blue represents the UC group; red represents the Control group. **(C)** The correlation between the hub gene and the immune cell. Blank area means that the significant level is higher than 0.05. *p < 0.05, **p < 0.01, and ***p < 0.001.

The CIBERSORT algorithm determined the infiltration of immune cells of UC. The differences in immune cell abundance between the UC group and healthy controls were evaluated, and samples with *p* > 0.05 were filtered. The correlation between the 22 immune cells is shown in **Figure 7A**. Activated mast cells and resting NK cells had the highest positive correlation (*r* = 0.58). Follicular helper T cells and naïve B cells also revealed a positive correlation (*r* = 0.51). Meanwhile, there was a negative correlation between follicular helper T cells and M2 macrophages, resting mast cells, and activated mast cells (*r* = −0.52). **Figure 7B** demonstrates the difference in the abundance of immune cell infiltration between the UC and the healthy control groups. The levels of CD4 memory activated T cells (*p* < 0.001), follicular helper T cells (*p* < 0.01), M0 macrophages (*p* < 0.001), M1 macrophages (*p* < 0.01), activated dendritic cells (*p* < 0.001), activated mast cells (*p* < 0.01), eosinophils (*p* < 0.05), and neutrophils (*p* < 0.01) were significantly higher in the UC group than in healthy controls. On the other hand, healthy controls showed higher levels of activated NK cells (*p* < 0.001), M2 macrophages (*p* < 0.001), and resting mast cells (*p* < 0.001). Moreover, CD4 memory activated T cells, follicular helper T cells, M1 macrophages, and neutrophils positively correlated with all the hub genes. Among them, neutrophils had the highest correlation (**Figure 7C**). M2 macrophage negatively correlated with all the hub genes (*p* < 0.05). *AIM2* showed the highest negative correlation with M2 macrophage, and *IL1B* and *TREM1* depicted the highest positive correlation with neutrophils. These results indicated that a severe immune imbalance occurred in the colon of UC patients, and the relationship between the different types of macrophages and UC was notable.

### Immune cell scRNA analysis of colonic lamina propria in inflamed UC

**Figure 8:**
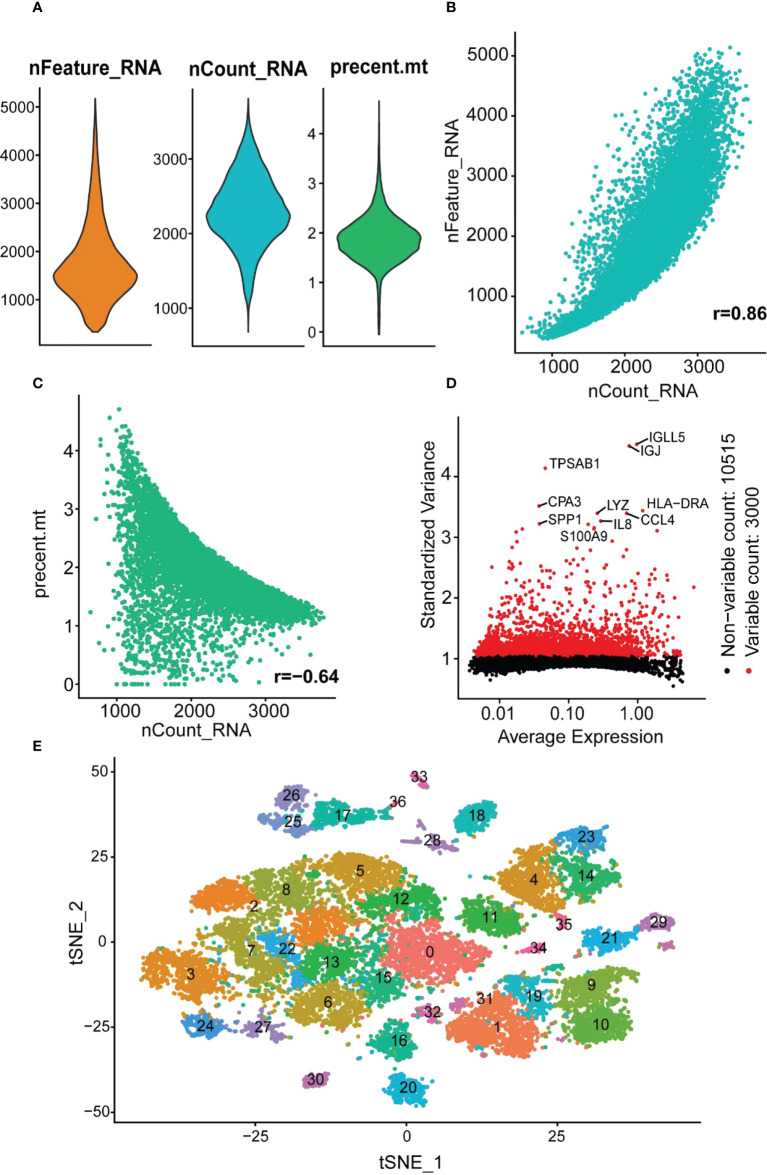
scRNA analysis of colonic lamina propria within inflamed UC. **(A)** The genes (features), counts, and mitochondrial gene percentages of the sample. **(B)** Correlation between genes and counts in the sample. **(C)** Correlation between genes and mitochondrial gene percentage in the sample. **(D)** The gene scatter plot with the top 10 HVGs. The red dots represent the HVGs, and the black dots represent other genes. **(E)** t-SNE projection of 18,375 immune cells, clustered into 37 classes.

The GSE162335 dataset was utilized for single-cell analysis. After filtering, 18,375 immune cells from the colonic lamina propria of inflamed UC patients were collected. The expression characteristics of the sample are shown in **Figure 8**. We normalized the data and identified 3,000 HVGs using “VST,” of which the top 10 HVGs were shown in **Figure 8D**. Principal component analysis and heatmap of the top 10 PCs with signature genes are shown in **Figure S5**. Thirty-seven were identified using the t-distributed stochastic neighbor embedding (t-SNE) method (**Figure 8E**), and the marker gene for each cell cluster is available in **Supplementary Table S6**.

**Figure 9:**
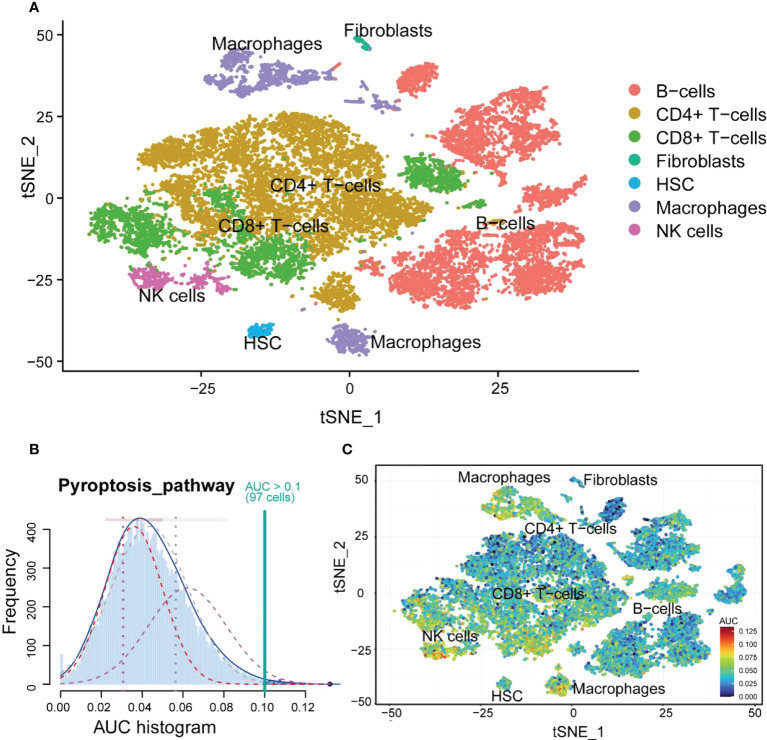
The cell annotation results and PRG scores of colonic lamina propria immune cell types in inflamed UC. **(A)** The results of cell annotation with different immune cell types are distinctively colored. **(B)** A score of 74 screened PRGs. The threshold was chosen as 0.10. **(C)** t-SNE plots of RPG score in all the cell types. Macrophages and NK cells express more genes and exhibit higher AUC values.

Seven immune cells were identified using different annotation methods, including B cells, CD4+ T cells, CD8+ T cells, fibroblasts, hematopoietic stem cells (HSCs), macrophages, and NK cells (**Figure 9A**). B cells and CD4+ T cells were the most numerous, while macrophages were significantly divided into two clusters.

### Macrophages highly correlated with pyroptosis

First, the relationship between immune cells and PRGs was explored, and we found that NK cells and macrophages had high AUC values (**Figures 9B, C**). Combining with the previous immune infiltration analysis results, we hypothesized that macrophages could be more closely related to pyroptosis. Therefore, we performed a KEGG analysis of DEGs in macrophages. The results showed that DEGs were mainly enriched in inflammatory diseases and signaling pathways and involved phagosome and lysosome (**Figure S6**).

**Figure 10:**
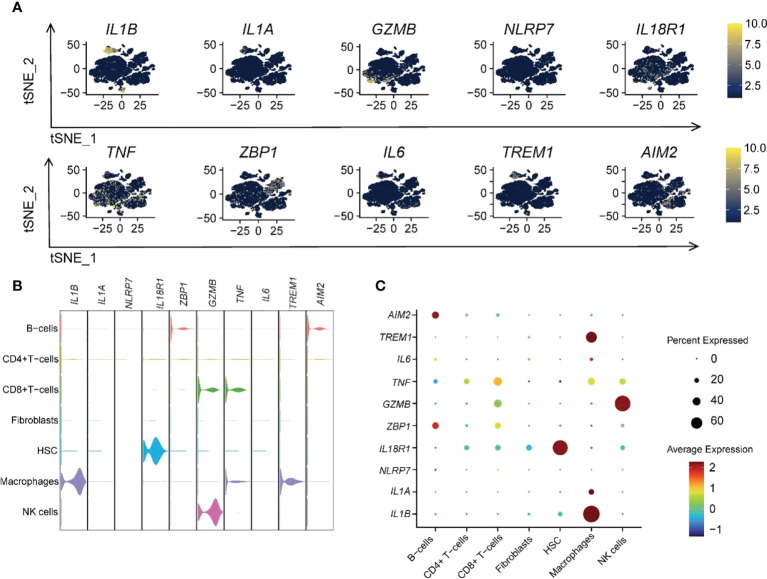
Ten RPGs hub gene expression patterns in different immune cells. **(A)** The t-SNE plot shows the distribution of hub genes. **(B)** The violin plot shows the expression levels of hub genes. **(C)** The dot plot depicts the expression patterns of hub genes.

We also analyzed the expression distribution of 10 hub genes within various immune cells (**Figure 10A**). The results revealed that *TNF* was more widely distributed, and *NLRP7* was less distributed among the seven immune cells. Almost all hub genes were expressed in macrophages, especially *IL1B*, the more critical hub gene, which was strongly expressed (**Figures 10B, C**). This indicated that macrophages could have a vital role in pyroptosis. Similarly, the high expression of *GZMB* in NK cells was also significant.

### Pseudotime analysis and enrichment analysis identification of pyroptosis related-macrophages clusters in UC

**Figure 11:**
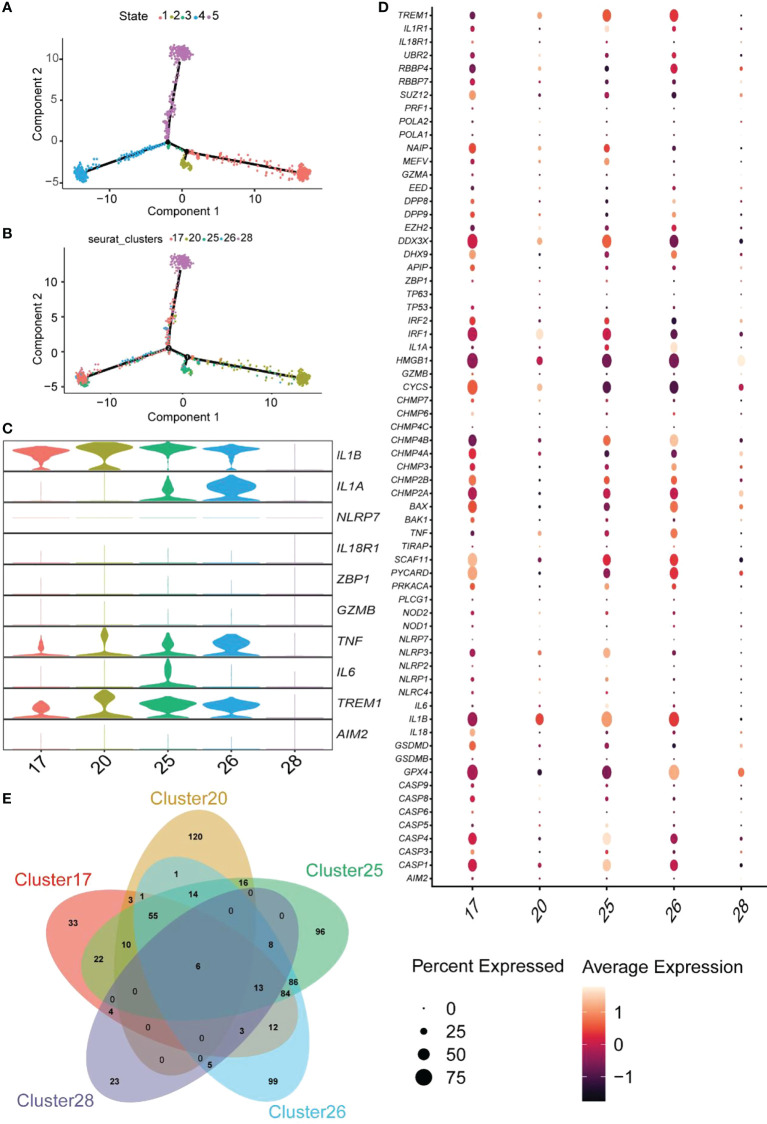
Pseudotime analysis of the clusters and expression patterns of PRG hub genes among different macrophage clusters. **(A, B)** Monocle pseudotime trajectory shows the progression of macrophages. **(C)** The violin plot demonstrates the expression levels of hub genes in five cluster macrophages. **(D)** The dot plot shows the expression pattern of 74 RPGs in five cluster macrophages. **(E)** The Venn diagram depicts the DEGs within each cluster macrophage.

We performed a pseudotime analysis of the five macrophage clusters, including clusters 17, 20, 25, 26, and 28, to reveal the relationship between macrophages and pyroptosis. The research showed that almost all cells were projected onto two branches of a trunk, where clusters 17, 20, and 28 were located at one pole each, and clusters 25 and 26 were on the evolutionary line (**Figures 11A, B**). Based on the expression of pyroptosis-related hub genes, clusters 17 and 20 were defined as *IL1B* ^+^ *IL1A* ^-^ *IL6* ^-^, cluster 25 was described as *IL1B* ^+^ *IL1A* ^+^ *IL6* ^+^, cluster 26 was designated as *IL1B* ^+^ *IL1A* ^+^ *IL6* ^-^, and cluster 28 was defined as the *IL1B* ^-^ *IL1A* ^-^ *IL6* ^-^ cell group (**Figure 11C**). The expression of 74 RPGs in five cellular clusters was assessed (**Figure 11D**). Almost all the RPGs were not expressed in cluster 28, which also predicted that cluster 28 was not related to pyroptosis. Clusters 17 and 20 had similar hub gene expression patterns, but cluster 17 expressed more RPGs than cluster 20.

GO enrichment analysis of unique DEGs from each cluster was performed to determine the molecular characteristics of the macrophages related to pyroptosis (**Supplementary Table S7**). Clusters 17, 20, 25, and 26 identified 33, 120, 96, and 99 DEGs (**Figure 11E**). GO terms of BP are shown in **Figure S7**. Enrichment analysis of cluster 17 indicated that this cluster was involved in several regulatory processes, including the positive regulation of immune response, cell adhesion regulation, and the immune system process (**Figure S7A**). Therefore, we named cluster17 as “Immunomodulatory macrophages.” The GO terms in cluster 20 focused on the negative regulatory processes of cells, and cluster 20 was termed “negative regulation macrophages” (**Figure S7B**). The trajectory analysis of cluster25 revealed that it was located between cluster 17 and 20, and enrichment analysis showed that it was mainly focused on the response process to external stimuli and immunity; thus, it was named “response macrophages” (**Figure S7C**). Finally, cluster26 was named a “transporter and secretory macrophages” (**Figure S7D**). These results indicated that macrophages related to pyroptosis could be classified into four types and perform different functions in the colonic lamina propria of inflamed UC patients. Therefore, exploring the relationship between macrophages and pyroptosis may facilitate a better understanding of pyroptosis occurrence in UC.

### Animal experiments to verify hub gene expression levels

**Figure 12:**
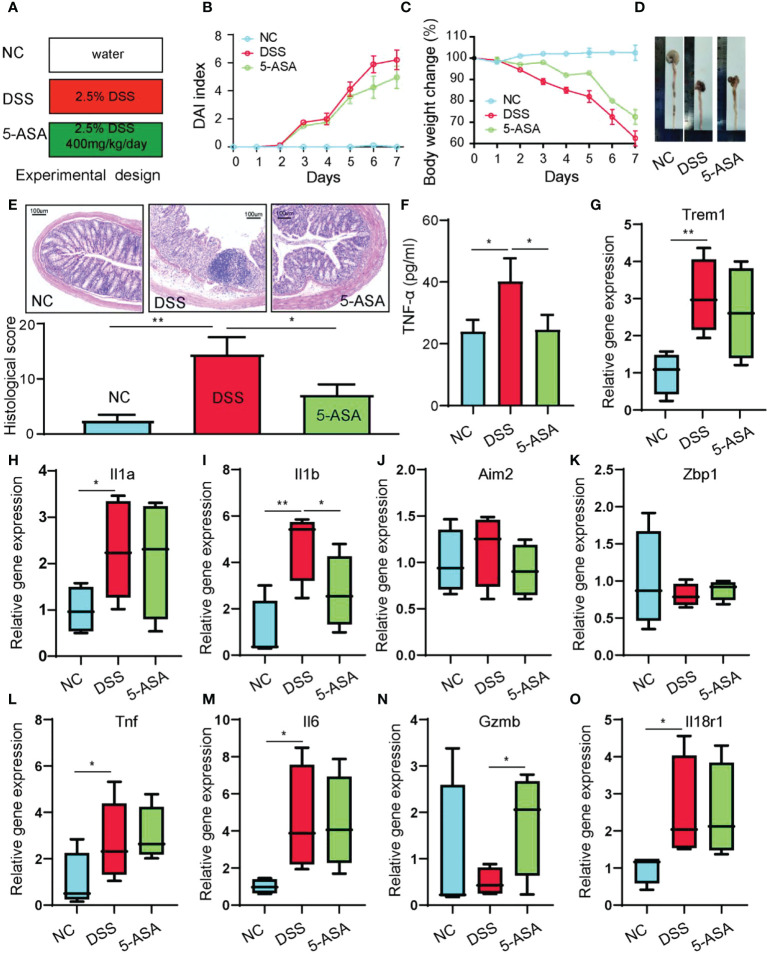
Animal experiments to verify the hub gene expression levels. **(A)** Experimental design. **(B)** DAI index. **(C)** Body weight change. **(D)** Length of colons. **(E)** H&E-stained colon tissue. **(F)** Serum TNF-α was determined with ELISA. The relative gene expression levels of *Trem1* **(G)**, *Il1a* **(H)**, *Il1b* **(I)**, *Aim2* **(J)**, *Zbp1* **(K)**, *Tnf* **(L)**, *Il6* **(M)**, *Gzmb* **(N)**, and *Il18r1* **(O)** were determined with RT-PCR. *p < 0.05, **p < 0.01.

The DSS-induced acute colitis was utilized to verify the gene expression of hub genes (**Figure 12A**). The results indicate that DSS-induced colitis in mice with bloody stools was relieved using 5-ASA treatment (**Figure 12B**). DSS-induced colitis caused weight loss and shorter colonic length in mice, and 5-ASA could suppress the weight loss and colon shortening (**Figures 12C, D**). The symptoms commonly occurring during UC, including bloody stools and shortening of the colon, are evidence of enhanced abnormal death of IECs. We performed histological staining and examination of colon sections of mice to observe the damage of intestinal mucosa during UC. Compared with the NC group, we found a series of features such as disorganized mucosal structure, inflammatory cell infiltration, crypt detachment, and IEC death in colon sections of mice in the DSS group. In contrast, 5-ASA treatment showed a protective effect on colonic mucosal structures (**Figure 12E**). There is strong evidence that UC is highly correlated with the death of IECs and intestinal barrier disruption, and therapeutic drugs can alleviate these symptoms. Therefore, inflammatory factor (TNF-α) in serum of DSS-induced mice was increased, while 5-ASA intervention decreased the elevation of TNF-α (**Figure 12F**). **Figures 12G–O** show that the relative expression levels of hub genes in DSS-induced colitis mice are consistent with bioinformatics analysis. The expression of most genes was elevated in the DSS group. Genes with inconsistent expression could be due to the species differences between mice and humans; *NLRP7* is associated with intestinal diseases such as colon cancer (33), but only a human-specific gene is not found in rodents (34). *ZBP1*, a critical innate sensor that recognizes and binds Z-RNA structures produced by various viruses, triggers different forms of cell death (35). However, microorganisms with such structures could be lacking in the pathogenic factors of DSS-induced colitis mice (36). A further outcome was that 5-ASA significantly downregulated only one gene, *IL1B*, without any significant effect on other genes. It suggests that there may be no relationship between the relief of colitis by 5-ASA and the regulation of pyroptosis, consistent with our previous findings.

## Discussion

UC is a common chronic inflammatory intestinal disease. The life quality of UC patients is severely impaired due to its recurrent and incurable characteristics (37). Although some progress has been made in treating UC, further mitigation of recurrence and eradication remains a global challenge. Pyroptosis, a new form of cell death discovered recently, has shown great promise in UC (38). For example, a study claims that DSS potentiates NLRP3 inflammasome activation by regulating the KCa3.1 potassium channel in a mouse model of colitis. This provides direct evidence for the role of pyroptosis in UC (39). In another study, the authors observed that Trans-10-Hydroxy-2-Decanoic acid could treat UC by inhibiting pyroptosis while also enhancing the barrier function of the colon (40). However, there is still a lack of reports concerning the detailed mechanism of pyroptosis in UC. Our study explores the role of pyroptosis in UC by identifying the relevant hub genes and immune cells. This will provide a new perspective on the pathogenesis and treatment of UC.

A total of 1,247 DEGs and 1,186 module genes were obtained using differential analysis and WGCNA. The enrichment analysis revealed that these genes were primarily involved in pyroptosis-related inflammatory pathways, including the NF-kappa B signaling pathway, the NOD-like receptor signaling pathway, the Toll-like receptor signaling pathway, and the TNF signaling pathway. This suggested that there is a close relationship between UC and pyroptosis. Subsequently, 10 pyroptosis-related hub genes (*AIM2*, *IL6*, *IL1B*, *NLRP7*, *TNF*, *IL1A*, *IL18R1*, *ZBP1*, *GZMB*, and *TREM1*) were identified. *AIM2* is a cytoplasmic sensor of double-stranded DNA from pathogens or damaged organelles. It recruits ASC and caspase-1 to develop the *AIM2* inflammasome, activating caspase-1 and processing IL-1β and IL-18, leading to pyroptosis (41). Innate immune sensor Z-DNA binding protein 1 (*ZBP1*) is the apical sensor of fungal infection and controls the NLRP3 inflammasome to participate in pyroptosis (42). It has been reported that *AIM2* in complex with pyrin and *ZBP1* can simultaneously engage in pyroptosis, apoptosis, and necroptosis, which is probably a novel research direction (43). *IL1B*, *IL1A*, *IL6*, and *TNF* are important pro-inflammatory cytokines in pyroptosis (44). *IL18R1* is the receptor for pro-inflammatory cytokine IL18 and is responsible for the binding of IL18 (45). The interleukin-1 cytokine family plays a crucial role in maintaining intestinal barrier integrity and inflammatory responses, but their specific functions are controversial. Various studies indicate their dual role in inflammation and homeostasis *in vivo* (46). *GZMB* catalyzes the cleavage of gasdermin-E (GSDME) and releases the pore-forming fraction of GSDME, thereby triggering pyroptosis (47). *TREM1* amplifies inflammatory signals involving Toll-like and NOD-like receptors, which mediates the exacerbation of pyroptosis (48). *NLRP7* is associated with innate immune signaling and affects macrophage polarization, but its exact role is unclear. In recent research, different pathways of pyroptosis have been identified (49). However, the specific mechanism of pyroptosis in UC remains poorly understood. Based on the 10 hub genes identified, we hypothesized that there could be two main pyroptosis pathways in UC. Firstly, the *AIM2* inflammasome-mediated caspase-1-dependent classical pyroptosis pathway was developed after dsDNA recognition by *AIM2* (50), and the caspase-independent pyroptosis pathway was controlled by *GZMB* (51). *AIM2*, *TREM1*, IL1B, *GZMB*, *IL18R1*, and *ZBP1* were diagnostic biomarkers. The results of ROC analysis indicated that *IL1B* has the most significant potential as a biomarker.

We observed the expression patterns of hub genes within the lesional/nonlesional, active/inactive/control groups by different datasets. All the results showed significant differences except for inactive/control groups, and among the inactive/control groups, *IL1B* became the only hub gene with significant differences. IL-1β is a potent pro-inflammatory factor that plays a crucial role in pyroptosis. The synthesis of pro-IL-1β is stimulated in response to an inflammatory signal. Then, when many inflammatory vesicles activate caspase-1, pro-IL-1β is cleaved to synthesize IL-1β, and subsequently, caspase-1 cleaves the GSDMD to create the 22-kDa C-terminus (C-GSDMD) and the 31-kDa N-terminus (N-GSDMD). N-GSDMD completes by forming pores in the plasma membrane and mitochondria to release IL-1β and IL-18. Thus, *IL1B* is a critical part of pyroptosis. Many reports have revealed that targeting *IL1B* can effectively alleviate UC (52). Combined with the previous ROC analyses, we speculate that *IL1B* is the most crucial gene in the pyroptosis of UC.

A characteristic feature of UC is that 80%–90% of patients have alternated between active and inactive intervals (53). The significant difference between the active and inactive phases of the hub gene could indicate the elevated expression of the hub gene in patients, causing damage to the intestinal mucosa triggered by pyroptosis in the IECs and an increase in pro-inflammatory factors, leading to recurrent disease (54). Thus, focusing on pyroptosis development is probably a new way of avoiding the recurrence of UC and a crucial future treatment direction. Moreover, we also explored the effects of some common UC drugs on hub genes to reveal the significance of pyroptosis in UC better. 5-ASA and biologics are common UC drugs but showed different effects on the hub gene. 5-ASA reduced the expression levels of *IL1B*, *IL1A*, and *IL6* but did not affect other hub genes. Biologics such as IFX and VDZ significantly decreased all hub genes. For 30 years, 5-ASA has been the treatment of choice against mild UC (55). However, a recent study showed that compared to biologic agents such as IFX and VDZ, 5-ASA did not help treat patients with moderate to severe UC, especially in controlling inflammation (56). Therefore, we speculated that the progression of UC disease could be associated with the degree of pyroptosis, and patients with severe UC may be subjected to a higher degree of intestinal mucosa invasion due to pyroptosis. Therefore, 5-ASA is not effective in treating patients having severe UC. In conclusion, we verified that both UC drugs had a different effect on the pyroptosis-related hub gene and decreased its expression while alleviating UC. The link between pyroptosis and the progression of UC deserves in-depth exploration. The expression of relevant genes/proteins needs to be verified by using the tissue from the patients. Reviewing the research literature with UC clinical samples, many previous studies reported the test results of the hub genes in patient tissues. For instance, IL-1β in serum and tissues of UC patients was measured by ELISA, and the results depicted that the expression of IL-1β in UC patients was significantly higher than in healthy controls (57, 58). In addition, the flow cytometry analysis revealed that the number of cells expressing GZMB and TREM1 in the intestinal mucosa of UC patients significantly increased (59, 60). Using Western blot analysis, the expression of AIM2 in active UC is higher than in inactive UC (28). These are consistent with the results from our bioinformatics analyses.

An immune infiltration analysis was performed to explore the dysregulation of inflammatory cells in UC. The results showed that immune cells were severely dysregulated within the colonic tissue of UC patients. Neutrophils, M0 macrophages, M1, activated dendritic cells, CD4 memory resting T cells, and follicular helper T cells were positively associated with hub genes. Single-cell analysis showed that nine hub genes were distributed in macrophages except for *NLRP7*, and *IL1B* was strongly expressed. In addition, macrophages were identified by AUCell evaluation to deliver the highest degree of response to the pyroptosis gene set. By enhancing the resolution, we identified five clusters of macrophages. Four clusters were closely associated with pyroptosis and may perform different biological functions. The pro-inflammatory and anti-inflammatory functions of macrophages have a crucial role in the development of UC, and many drugs alleviate UC by targeting the macrophages (61, 62). However, the interaction between different immune cell types requires further exploration. More importantly, the alleviation of UC by inhibiting macrophage pyroptosis has been described in recent years (15). It was also shown that the lactic acid-producing probiotic *Saccharomyces cerevisiae* inhibits the hyperactivation of the NLRP3 inflammasome and downstream caspase-1 pathway among macrophages, thereby inhibiting pyroptosis. When macrophage pyroptosis was inhibited, the colonic mucosal barrier was strengthened, the immune response in the intestine decreased, and histological damage was restored.

Abnormal death of IECs causes damage to the intestinal mucosa, and this structural weakening of the intestinal mucosal barrier is an early event in UC pathogenesis (54). Pyroptosis is a necessary form of death for IECs. A series of pro-inflammatory factors secreted during pyroptosis, including IL-18 and IL-1β, can disrupt the integrity of the intestinal mucosal barrier (63). Therefore, excessive death of IECs and the secretion of pro-inflammatory factors due to excessive activation of pyroptosis are essential factors in disrupting the intestinal barrier, which could be a critical mechanism in UC pathogenesis. After disrupting the intestinal barrier, microorganisms in the gut invade the intestine, causing a series of immune responses while macrophages undergo pyroptosis. The pro-inflammatory factors released in pyroptosis could worsen the immune response in the intestine and lead to more death of IECs, further disrupting the intestinal barrier. This may be one of the reasons why UC is incurable and recurrent.

## Conclusion

Ten pyroptosis-related hub genes in UC were identified, and the expression pattern of hub genes was validated. The effect of the existing UC treatment drugs on the gene expression of hub genes was explored, and *IL1B* was identified as the predictor for drug response and a marker for the active state of UC. Combining single-cell analysis and immune infiltration, we identified macrophages as the most relevant immune cell type in UC progression. Our study explored the molecular mechanisms of the pyroptosis process in UC. We proposed that the crosstalk between macrophages and IECs regarding pyroptosis could be responsible for the incurability and recurrence of UC. The *IL1B*–macrophage–pyroptosis relationship chain provides a new perspective on the pathogenesis and treatment of UC.

## Data availability statement

The original contributions presented in the study are included in the article/**Supplementary Material**. Further inquiries can be directed to the corresponding authors.

## Ethics statement

This study was reviewed and approved by Ethics Committee Medical College of Qingdao University (QDU-AEC-2022314).

## Author contributions

NH and SL conceived the study and performed data analysis. KC and SS collected data and performed data analysis. SY and LC performed animal experiments and related tests. NH and SL supervised the project. KC, SS, SL and NH wrote the manuscript. All authors contributed to the article and approved the submitted version.

## Funding

This study was supported by the Shandong Provincial Youth Entrepreneurship Program for Colleges and Universities (2021KJ075) and National Natural Science Foundation of China (NSFC) [grant number 31900031] and Shandong Provincial Natural Science Foundation [grant number ZR2019BD027].

## Acknowledgments

We sincerely thank Professor Shumei Zhang from College of Information and Computer Engineering of Northeast Forestry University for reviewing the statistical methods in this study.

## Conflict of interest

The authors declare that the research was conducted in the absence of any commercial or financial relationships that could be construed as a potential conflict of interest.

## Publisher’s note

All claims expressed in this article are solely those of the authors and do not necessarily represent those of their affiliated organizations, or those of the publisher, the editors and the reviewers. Any product that may be evaluated in this article, or claim that may be made by its manufacturer, is not guaranteed or endorsed by the publisher.
