# Supplementary material for: Identification and exploration of pharmacological pyroptosis-related biomarkers of ulcerative colitis
Source: Front Immunol. 2022 Oct 13;13:998470. doi: 10.3389/fimmu.2022.998470 (PMC9606687; doi:10.3389/fimmu.2022.998470)
Supplement: Supplementary file 1 [file DataSheet_1.zip › Supplementary Material 3.PDF]

Supplementary Material 3 : Characteristics of patients and controls in GSE59071

*: immunosupperessants = azathioprine/6-mercaptopurine and methotrexate, IQR: interquartile range, IFX: infliximab, NA: not applicable

**Characteristics**	UC		CD	Controls
	Active	Inactive	Active	
	(n=74)	(n=23)	(n=8)	(n=11)
**Male/Female (%)**	43/31 (58.1/41.9)	12/11 (52.2/47.8)	2/6 (25/75)	5/6 (45.5/54.5)
**Median (IQR)* age (years)**	45.9 (33.19-54.97)	43.93 (29.6-57.2)	38.9 (35.1-45.3)	69.6 (64-74.9)
**Median (IQR)* duration of disease (years)**	5.46 (2.59-13.5)	8.65 (3.3-17.9)	2 (0,19-3.99)	NA
**Extent of disease**				
**UC Left-sided colitis (%)**	35 (47.3)	13 (56.5)	NA	NA
**Pancolitis (%)**	39 (52.7)	10 (43.5)	NA	NA
**CD Ileocolon (%)**	NA	NA	3 (37.5)	NA
**Ileum (%)**	NA	NA	0 (0)	NA
**Colon (%)**	NA	NA	5 (62.5)	NA
**Concomitant medication at first IFX (%)**				
**5-Aminosalicylates**	59 (79.7)	22 (95.7)	1 (12.5)	NA
**Corticosteroids**	31 (41.9)	2 (8.7)	2 (25)	NA
**Azathioprine/6-Mercaptopurine**	13 (17.6)	10 (43.5)	0 (0)	NA
**Methotrexate**	2 (2.7)	0 (0)	0 (0)	NA
**Anti-TNF**	0 (0)	11 (47.8)	0 (0)	NA
**Active smoking (%)**	9 (12.2)	3 (13)	3 (37.5)	0 (0)
**Azathioprine/6-Mercaptopurine**	13 (17.6)	10 (43.5)	0 (0)	NA
**Methotrexate**	2 (2.7)	0 (0)	0 (0)	NA
**Anti-TNF**	0 (0)	11 (47.8)	0 (0)	NA
**Active smoking (%)**	9 (12.2)	3 (13)	3 (37.5)	0 (0)

Data obtained from Vanhove W, Peeters PM, Staelens D, Schraenen A, Van der Goten J, Cleynen I, et al. Strong Upregulation of Aim2 and Ifi16 Inflammasomes in the Mucosa of Patients with Active Inflammatory Bowel Disease. Inflammatory bowel diseases (2015) 21(11):2673-82. Epub 2015/08/28. doi: 10.1097/mib.0000000000000535.
