# Supplementary material for: Identification and exploration of pharmacological pyroptosis-related biomarkers of ulcerative colitis
Source: Front Immunol. 2022 Oct 13;13:998470. doi: 10.3389/fimmu.2022.998470 (PMC9606687; doi:10.3389/fimmu.2022.998470)
Supplement: Supplementary file 1 [file DataSheet_1.zip › Supplementary Material 4.PDF]

Supplementary Material 4

Baseline characteristics	UC VDZ (n=41)	UC IFX (n=23)	Controls (n=12)
Male/female (%)	21/20 (51.2/48.8)	13/10 (56.5/43.5)	6/6 (50/50)
Median (IQR) age (years)	40.5 (32–49.4)	41.3 (31.1–49.6)	68.2 (59–72.7)
Median (IQR) duration of disease (years)	10.2 (4.4–14.6)	7.6 (2.8–17.2)	NA
Extent of disease			
UC left-sided colitis/ pancolitis (%)	18/23 (43.9/56.1)	6/17 (26.1/73.9)	NA
Histology (Geboes score)			
0–1 (%)	0 (0)	0 (0)	NA
2–5 (%)	41 (100)	23 (100)	NA
Mayo endoscopic subscore			
0–1 (%)	0 (0)	0 (0)	NA
2–3 (%)	41 (100)	23 (100)	NA
Median (IQR) total Mayo score	10 (8–11)	10 (9–10)	NA
Medication (%)			
5-Aminosalicylates	29 (70.7)	18 (78.3)	NA
Corticosteroids	17 (41.5)	7 (30.4)	NA
Azathioprine/ 6-mercaptopurine	7 (17.1)	14 (60.8)	NA
Methotrexate	1 (2)	0 (0)	NA
Anti-TNF	0 (0)	0 (0)	NA
Active smoking (%)	5 (12.2)	2 (8.3)	0 (0)

IFX, infliximab; NA, not applicable; TNF, tumour necrosis factor; VDZ, vedolizumab.

Data obtained from Effect of Vedolizumab (Anti -A4β7-Integrin) Therapy on Histological Healing and Mucosal Gene Expression in Patients with Uc. Gut (2018) 67(1):43-52. Epub 2016/11/02. doi: 10.1136/gutjnl-2016-312293.
