# Supplementary material for: Identification and exploration of pharmacological pyroptosis-related biomarkers of ulcerative colitis
Source: Front Immunol. 2022 Oct 13;13:998470. doi: 10.3389/fimmu.2022.998470 (PMC9606687; doi:10.3389/fimmu.2022.998470)
Supplement: Supplementary file 1 [file DataSheet_1.zip › Supplementary Material 5.PDF]

Supplementary Material 5

Variable	**Normal pouch n=5**	**Pouchitis n=10**	**Ulcerative colitis n=13**
Sex			
Female	2 (40%)	4 (40%)	7 (54%)
Age at IBD diagnosis (median, IQR)	36 (30-38)	28 (13-37)	24 (19-27)
Maximum extent at IPAA or biopsy			
Proctitis (E1)	0	0	2 (15%)
Left-sided colitis (E2)	0	1 (10%)	3 (23%)
Extensive colitis (E3)	5 (100%)	9 (90%)	7 (54%)
Age at IPAA (median, IQR)	42 (39-45)	34 (16-40)	-
Indication for IPAA			-
Medically refractory disease	5 (100%)	9 (90%)	
Colorectal neoplasia	0	1 (10%)	
Previous medication exposures			
Systemic steroid	3 (60%)	9 (90%)	11 (85%)
Aminosalicylate	5 (100%)	10 (100%)	13 (100%)
Immunomodulator	2 (40%)	7 (70%)	2 (15%)
Anti-tumor necrosis factor	4 (80%)	8 (80%)	9 (69%)
Anti-integrin	2 (40%)	1 (10%)	8 (62%)
Anti- IL12/23	0	2 (20%)	0
Janus kinase inhibitor	0	1 (10%)	0
Antibiotic	5 (100%)	10 (100%)	7 (54%)
Medication exposure at biopsy			
Systemic steroid	0	3 (30%)	4 (31%)
Aminosalicylate	0	1 (10%)	5 (39%)
Immunomodulator	0	1 (10%)	0
Anti-tumor necrosis factor	0	1 (10%)	3 (23%)
Anti-integrin	0	0	5 (39%)
Anti- IL12/23	0	1 (10%)	0
Janus kinase inhibitor	0	1 (10%)	1 (8%)
Antibiotic	0	4 (40%)	0
Mayo score (median, IQR)	-	-	10 (10-10)
Partial			7 (7-7)
Endoscopic			3 (3-3)
PDAI (median, IQR)	1 (1-2)	6 (3-10)	-
Clinical	1 (1-2)	2 (0-4)	
Endoscopic	0	3 (2-4)	
Histologic	1 (1-1)	1 (1-2)	

Abbreviations: Inflammatory bowel disease (IBD); Ileal pouch-anal anastomosis (IPAA); Pouchitis disease activity index (PDAI).

Data obtained from Single-Cell Transcriptional Survey of Ileal-Anal Pouch Immune Cells from Ulcerative Colitis Patients. Gastroenterology (2021) 160(5):1679-93. Epub 2020/12/29. doi: 10.1053/j.gastro.2020.12.030.
