# Supplementary material for: Identification and exploration of pharmacological pyroptosis-related biomarkers of ulcerative colitis
Source: Front Immunol. 2022 Oct 13;13:998470. doi: 10.3389/fimmu.2022.998470 (PMC9606687; doi:10.3389/fimmu.2022.998470)
Supplement: Supplementary file 1 [file DataSheet_1.zip › FigureS5.pdf]

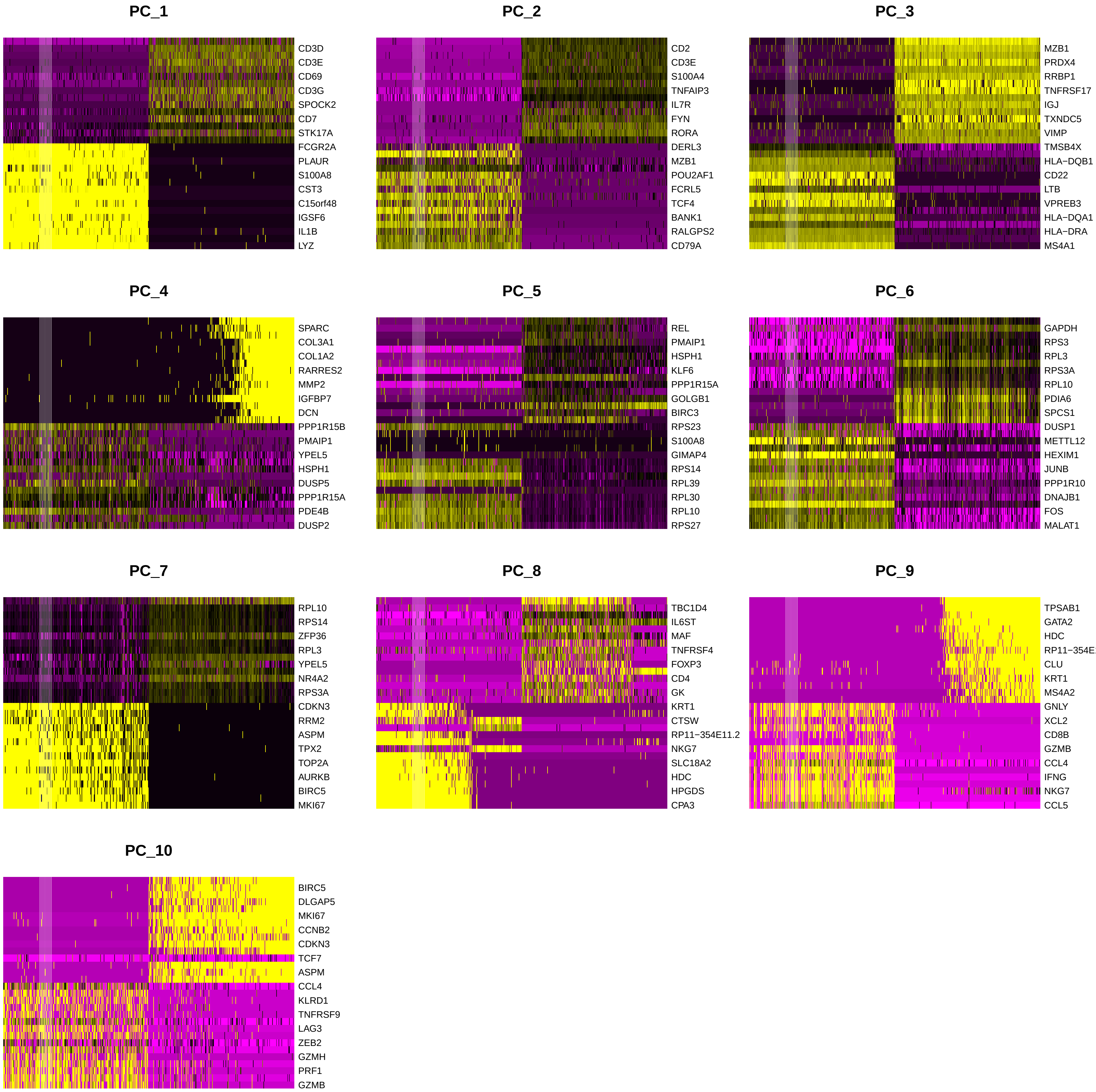
PC_1
CD3D
CD3E
CD69
CD3G
SPOCK2
CD7
STK17A
FCGR2A
PLAUR
S100A8
CST3
C15orf48
IGSF6
IL1B
LYZ
PC_2
CD2
CD3E
S100A4
TNFAIP3
IL7R
FYN
RORA
DERL3
MZB1
POU2AF1
FCRL5
TCF4
BANK1
RALGPS2
CD79A
PC_3
MZB1
PRDX4
RRBP1
TNFRSF17
IGJ
TXNDC5
VIMP
TMSB4X
HLA−DQB1
CD22
LTB
VPREB3
HLA−DQA1
HLA−DRA
MS4A1
PC_4
SPARC
COL3A1
COL1A2
RARRES2
MMP2
IGFBP7
DCN
PPP1R15B
PMAIP1
YPEL5
HSPH1
DUSP5
PPP1R15A
PDE4B
DUSP2
PC_5
REL
PMAIP1
HSPH1
KLF6
PPP1R15A
GOLGB1
BIRC3
RPS23
S100A8
GIMAP4
RPS14
RPL39
RPL30
RPL10
RPS27
PC_6
GAPDH
RPS3
RPL3
RPS3A
RPL10
PDIA6
SPCS1
DUSP1
METTL12
HEXIM1
JUNB
PPP1R10
DNAJB1
FOS
MALAT1
PC_7
RPL10
RPS14
ZFP36
RPL3
YPEL5
NR4A2
RPS3A
CDKN3
RRM2
ASPM
TPX2
TOP2A
AURKB
BIRC5
MKI67
PC_8
TBC1D4
IL6ST
MAF
TNFRSF4
FOXP3
CD4
GK
KRT1
CTSW
RP11−354E11.2
NKG7
SLC18A2
HDC
HPGDS
CPA3
PC_9
TPSAB1
GATA2
HDC
RP11−354E
CLU
KRT1
MS4A2
GNLY
XCL2
CD8B
GZMB
CCL4
IFNG
NKG7
CCL5
PC_10
BIRC5
DLGAP5
MKI67
CCNB2
CDKN3
TCF7
ASPM
CCL4
KLRD1
TNFRSF9
LAG3
ZEB2
GZMH
PRF1
GZMB
