# Supplementary material for: Identification and exploration of pharmacological pyroptosis-related biomarkers of ulcerative colitis
Source: Front Immunol. 2022 Oct 13;13:998470. doi: 10.3389/fimmu.2022.998470 (PMC9606687; doi:10.3389/fimmu.2022.998470)
Supplement: Supplementary file 1 [file DataSheet_1.zip › Supplementary Material 1.DOCX]

**The description information for datasets used in this study**

**GSE87466**

The molecular profile of adult UC was derived from a phase 3 clinical trial of golimumab in adults with moderate to severe UC (biopsy subgroup, n=87). Additional healthy control biopsies (n=21) were obtained from the Department of Gastroenterology, Perelman School of Medicine at the University of Pennsylvania (Philadelphia,PA) and the Department of Gastroenterology, University Hospital Gasthuisberg (Leuven,Belgium). The adult control biopsies were obtained from the colon without specification of colonic segment. In the clinical trials, endoscopists were instructed to obtain biopsies 15-20 cm from the anal verge from locations representative of the degree of inflammation seen in the region. More information is available in **Supplementary Material 2.**

**GSE92415**

Information about the subjects is available in **Supplementary Material 2.**

**GSE107499**

The study included a total of 44 non-lesional colon tissue and 75 lesional colon tissue from UC patients. Information about the subjects is available in **Supplementary Material 2**

**GSE59071**

The dataset contains colonic mucosal biopsies from 97 UC patients, and 11 normal mucosal controls. Biopsies were taken from the edge of the ulcers in the most inﬂamed part of the colon (sigmoid or rectum). Disease activity was endoscopically assessed. In UC,there were 74 patients with active disease (endoscopic Mayo sub-score 2–3) and 23 with inactive disease (endoscopic Mayo sub-score 0–1). All controls underwent endoscopy for screening ofpolyps and had an endoscopically normal mucosa. More information is available in **Supplementary Material 2** and **Supplementary Material 3.**

**GSE73661**

The study was carried out at the University Hospitals Leuven (Leuven, Belgium). The characteristics of patients and controls are summarised in **Supplementary Material 4.** A total of 44 UC patients about VDZ treatment were enrolled in the study, and 41 out of 44 patients were treated with VDZ at inclusion. Biopsies were taken at protocol- speciﬁed time points (week (W) 0, W6, W12 and W52, or at study withdrawal). A total of 120 colonic mucosal biopsies were available for analysis. As control groups, colonic mucosal biopsies were collected from 23 patients with UC before and W4–6 after first IFX therapy as well as from 12 non-IBD control individuals with normal mucosa. Biopsies were taken in the colon at the edge of ulcers when ever present. If no ulcers were seen, then biopsies were taken at the most inflamed colon segment. More information about the subjects is available in in **Supplementary Material 2.**

**GSE46451**

More information on the sample is available in **Supplementary Material 2.**

**GSE162335**

13 patients with UC were recruited and consented at the IBD Center under an NYU Langone Health IRB-approved study (S12-01137). Endoscopic appearance determined inflammatory activity. All UC patients (n=11) had a Mayo endoscopic subscore of 2. Typically, 6 pinch biopsies were obtained from each patient, however fewer biopsies were obtained from severely inflamed patients to reduce risk of excessive bleeding and perforation, which may have contributed to fewer recovered cells in inflamed samples. Cryopreserved biopsies were gently thawed at 37°C and enzymatically digested in collagenase VIII (Sigma) and DNase (Sigma) for 1h to obtain single cell suspensions for sorting CD45+ cells on a Sony SY3200 cell sorter (live/dead, CD45 PE-Cy7, CD3 PerCP-Cy5.5, CD19 PE, CD14 FITC, and CD16 Pacific Blue (BioLegend) for scRNA-seq. The demographic characteristics of these 11 patients can be obtained in **Supplementary Material 5.**
