# Supplementary figures and images for: Identification and exploration of pharmacological pyroptosis-related biomarkers of ulcerative colitis
Source: Front Immunol. 2022 Oct 13;13:998470. doi: 10.3389/fimmu.2022.998470 (PMC9606687; doi:10.3389/fimmu.2022.998470)

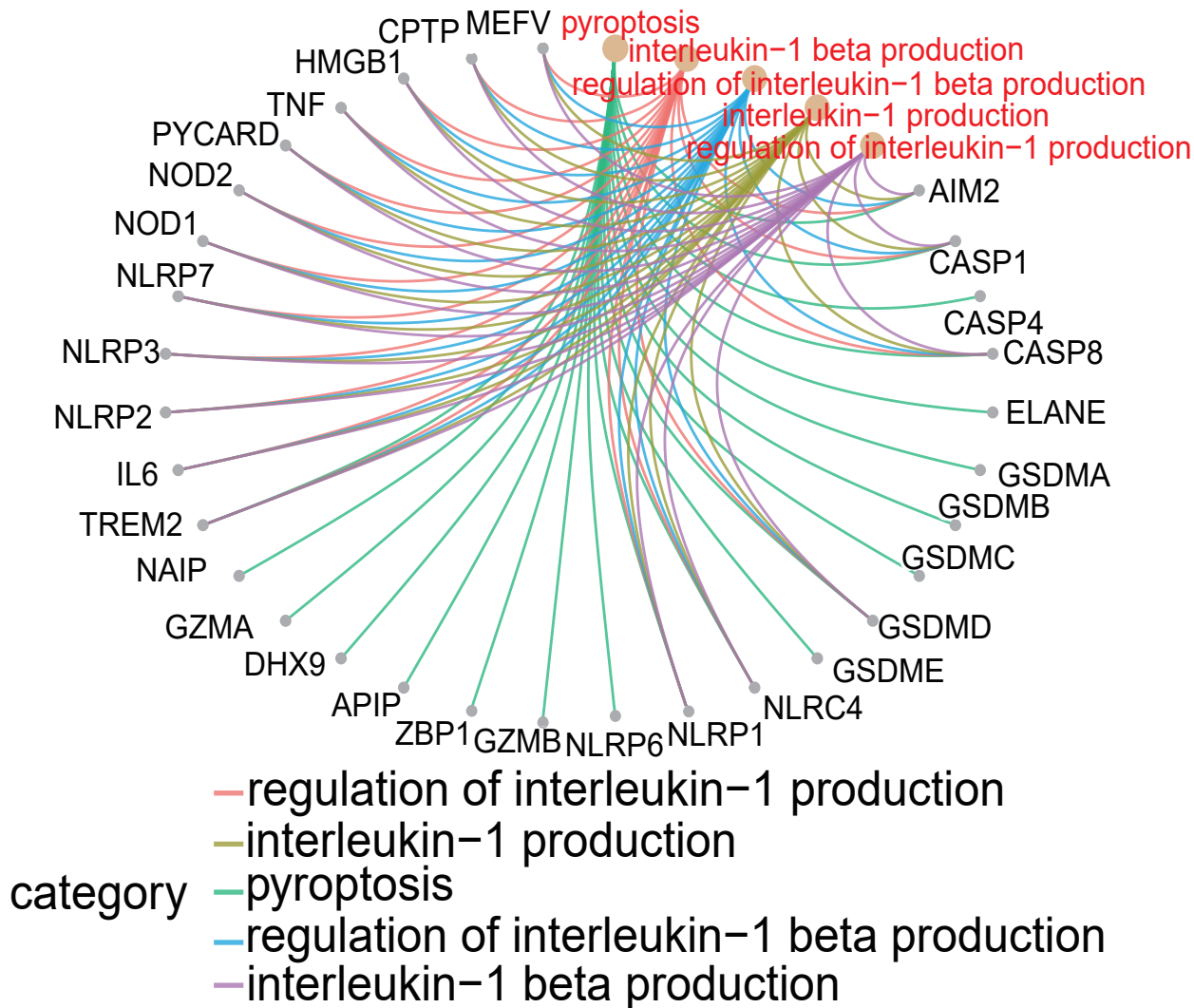

Supplement: Supplementary file 1 [file DataSheet_1.zip › FigureS2.pdf]

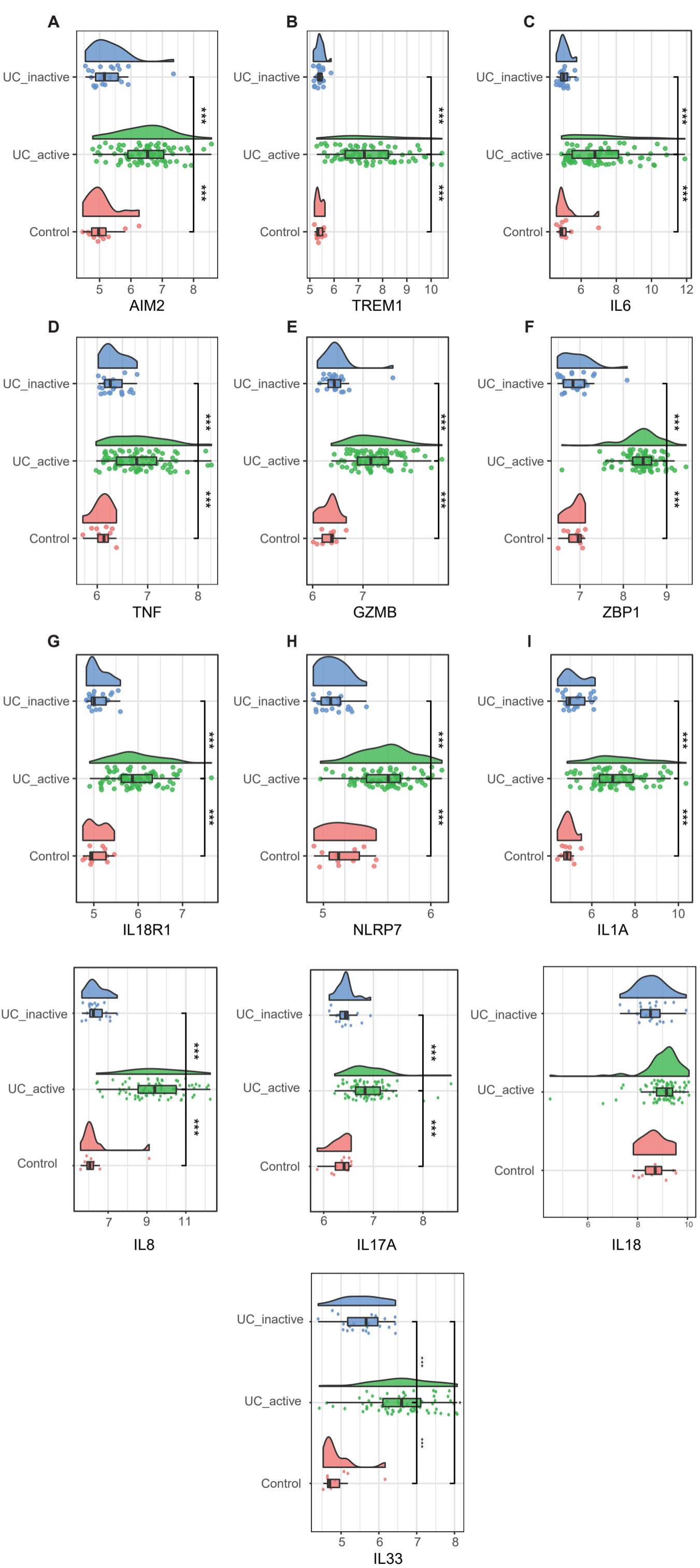

Supplement: Supplementary file 1 [file DataSheet_1.zip › FigureS3.pdf]

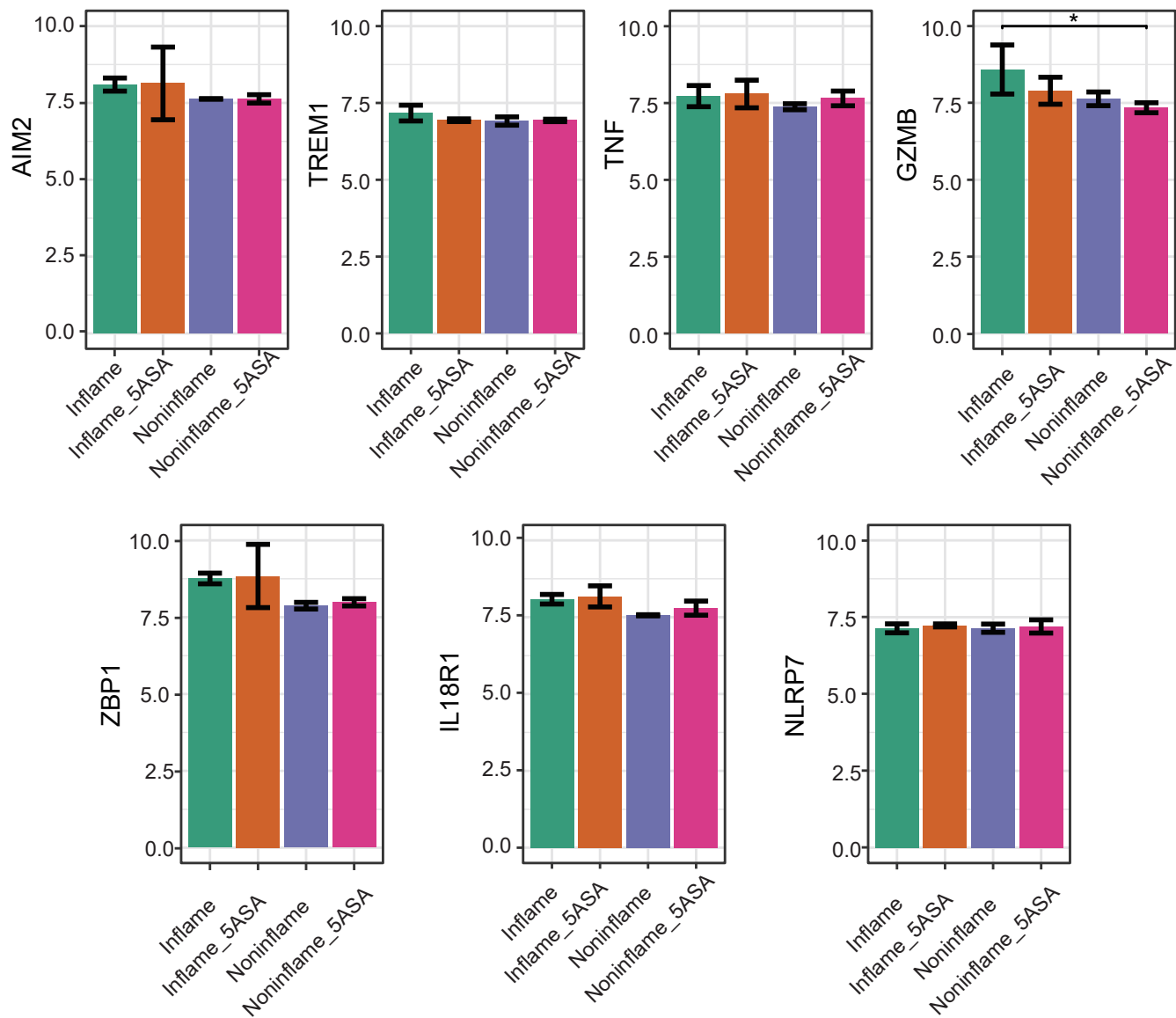

Supplement: Supplementary file 1 [file DataSheet_1.zip › FigureS4.pdf]

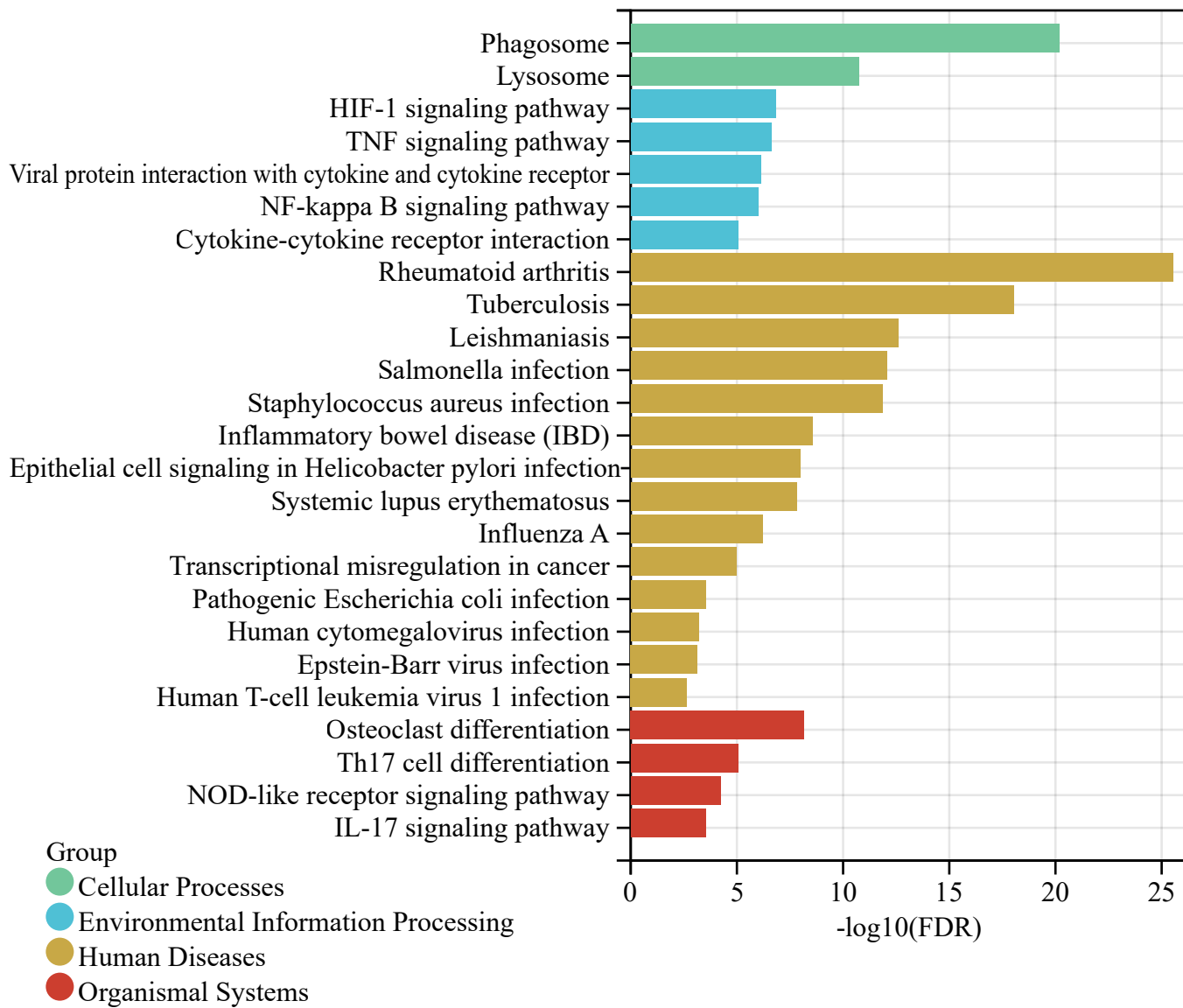

Supplement: Supplementary file 1 [file DataSheet_1.zip › FigureS6.pdf]
