# Supplementary material for: Identification and exploration of pharmacological pyroptosis-related biomarkers of ulcerative colitis
Source: Front Immunol. 2022 Oct 13;13:998470. doi: 10.3389/fimmu.2022.998470 (PMC9606687; doi:10.3389/fimmu.2022.998470)
Supplement: Supplementary file 1 [file FigureS1.pdf › FigureS1.pdf]

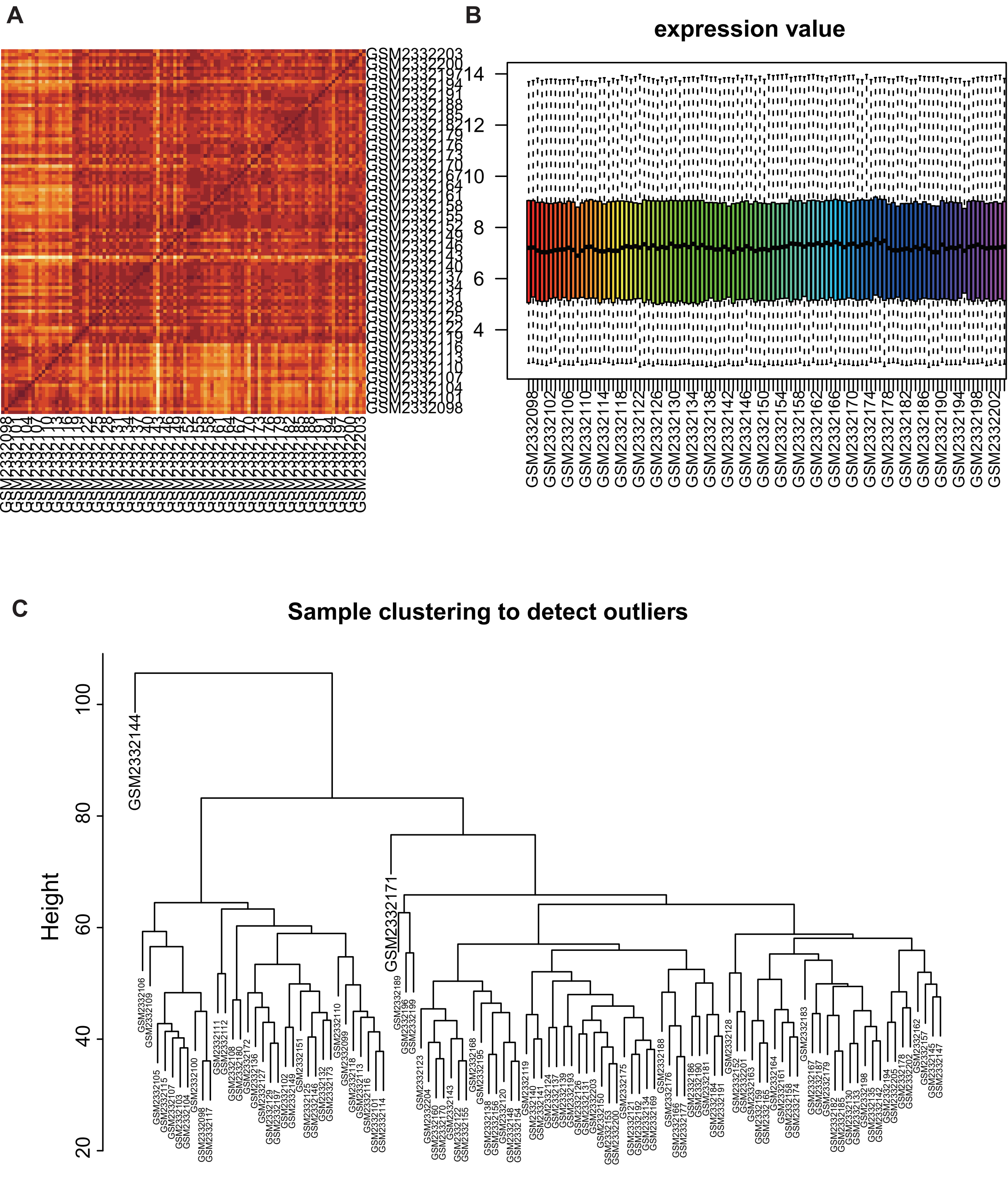
A
B
expression value
14
12
10
8
6
4
GSM2332098
GSM2332102
GSM2332106
GSM2332110
GSM2332114
GSM2332118
GSM2332122
GSM2332126
GSM2332130
GSM2332134
GSM2332138
GSM2332142
GSM2332146
GSM2332150
GSM2332154
GSM2332158
GSM2332162
GSM2332166
GSM2332170
GSM2332174
GSM2332178
GSM2332182
GSM2332186
GSM2332190
GSM2332194
GSM2332198
GSM2332202
C
Sample clustering to detect outliers
Height
100
80
60
40
20
GSM2332144
GSM2332171
