# Supplementary material for: Identification and exploration of pharmacological pyroptosis-related biomarkers of ulcerative colitis
Source: Front Immunol. 2022 Oct 13;13:998470. doi: 10.3389/fimmu.2022.998470 (PMC9606687; doi:10.3389/fimmu.2022.998470)
Supplement: Supplementary file 1 [file FigureS7.pdf › FigureS7.pdf]

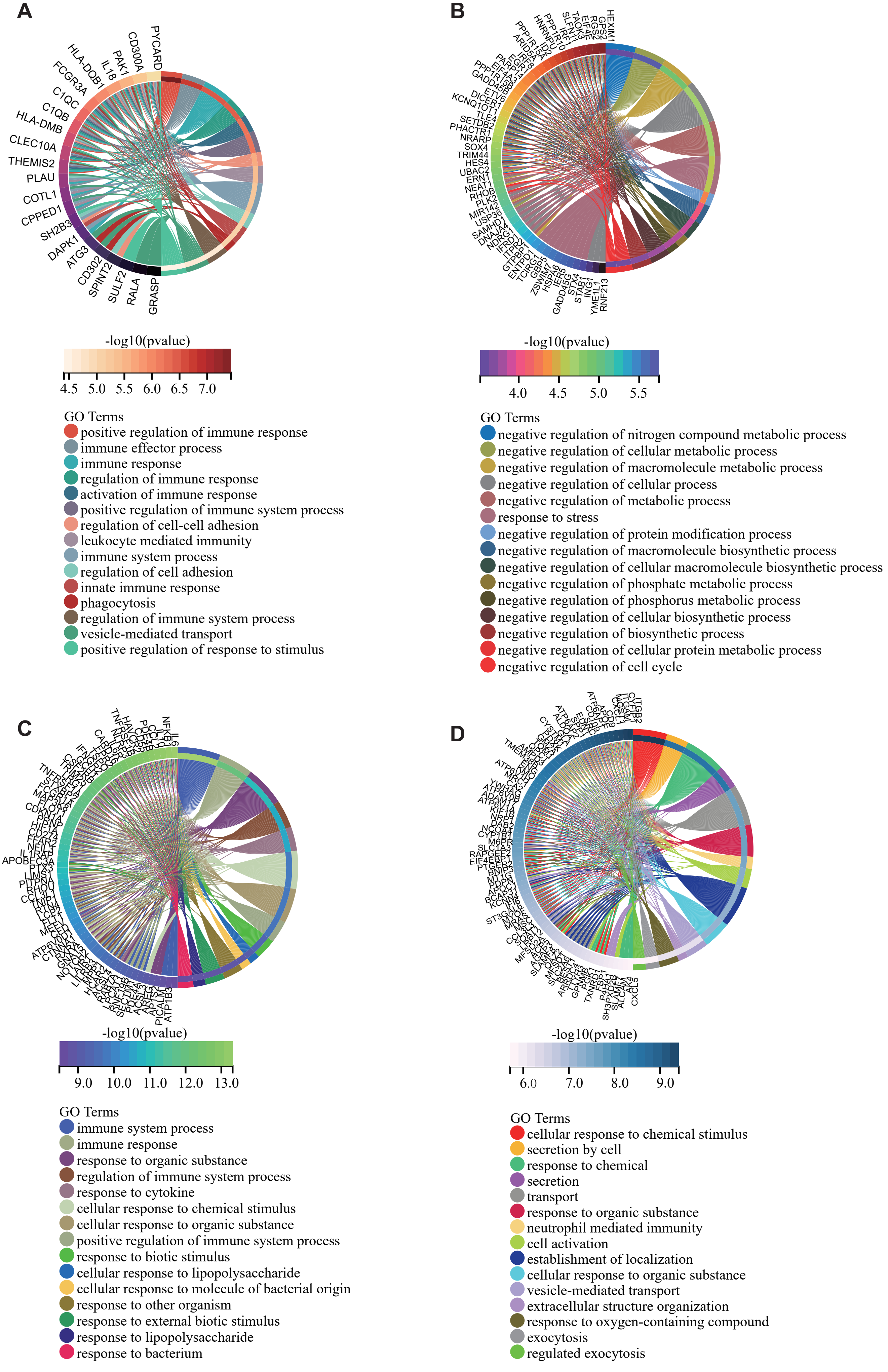
A
PYCARD
CD300A
PAK1
IL18
HLA-DQB1
FCGR3A
C1QC
C1QB
HLA-DMB
CLEC10A
THEMIS2
PLAU
COTL1
CPPED1
SH2B3
DAPK1
ATG3
CD302
SPINT2
SULF2
RALA
GRASP
-log10(pvalue)
4.5 5.0 5.5 6.0 6.5 7.0
GO Terms
positive regulation of immune response
immune effector process
immune response
regulation of immune response
activation of immune response
positive regulation of immune system process
regulation of cell-cell adhesion
leukocyte mediated immunity
immune system process
regulation of cell adhesion
innate immune response
phagocytosis
regulation of immune system process
vesicle-mediated transport
positive regulation of response to stimulus
B
HEXIM1
GPS2
RGS2
EIF4E
TAOK3
SLFN5
PPP1R10
HNRNPD
ARID5A
PPP1R15A
KCNQ1OT1
TLE4
SETDB2
PHACTR1
NRARP
SOX4
TRIM44
HES4
UBAC2
ERN1
NEAT1
RHOB
PLK2
MIR142
USP36
SAMHD1
DNAJA4
NDRG1
IFRD1
GTPBP1
ENTPD1
TCIRG1
GBP5
ZSWIM7
HSPA6
IER5
GADD45G
STX4
STAB1
ING1
YME1L1
RNF213
-log10(pvalue)
4.0 4.5 5.0 5.5
GO Terms
negative regulation of nitrogen compound metabolic process
negative regulation of cellular metabolic process
negative regulation of macromolecule metabolic process
negative regulation of cellular process
negative regulation of metabolic process
response to stress
negative regulation of protein modification process
negative regulation of macromolecule biosynthetic process
negative regulation of cellular macromolecule biosynthetic process
negative regulation of phosphate metabolic process
negative regulation of phosphorus metabolic process
negative regulation of cellular biosynthetic process
negative regulation of biosynthetic process
negative regulation of cellular protein metabolic process
negative regulation of cell cycle
C
NFKB1
APOBEC3A
PICALM
ATP1B3
-log10(pvalue)
9.0 10.0 11.0 12.0 13.0
GO Terms
immune system process
immune response
response to organic substance
regulation of immune system process
response to cytokine
cellular response to chemical stimulus
cellular response to organic substance
positive regulation of immune system process
response to biotic stimulus
cellular response to lipopolysaccharide
cellular response to molecule of bacterial origin
response to other organism
response to external biotic stimulus
response to lipopolysaccharide
response to bacterium
D
CXCL5
-log10(pvalue)
6.0 7.0 8.0 9.0
GO Terms
cellular response to chemical stimulus
secretion by cell
response to chemical
secretion
transport
response to organic substance
neutrophil mediated immunity
cell activation
establishment of localization
cellular response to organic substance
vesicle-mediated transport
extracellular structure organization
response to oxygen-containing compound
exocytosis
regulated exocytosis
